# Relationship of suicide rates with climate and economic variables in Europe during 2000–2012

**DOI:** 10.1186/s12991-016-0106-2

**Published:** 2016-08-09

**Authors:** Konstantinos N. Fountoulakis, Isaia Chatzikosta, Konstantinos Pastiadis, Prodromos Zanis, Wolfram Kawohl, Ad J. F. M. Kerkhof, Alvydas Navickas, Cyril Höschl, Dusica Lecic-Tosevski, Eliot Sorel, Elmars Rancans, Eva Palova, Georg Juckel, Goran Isacsson, Helena Korosec Jagodic, Ileana Botezat-Antonescu, Janusz Rybakowski, Jean Michel Azorin, John Cookson, John Waddington, Peter Pregelj, Koen Demyttenaere, Luchezar G. Hranov, Lidija Injac Stevovic, Lucas Pezawas, Marc Adida, Maria Luisa Figuera, Miro Jakovljević, Monica Vichi, Giulio Perugi, Ole A. Andreassen, Olivera Vukovic, Paraskevi Mavrogiorgou, Peeter Varnik, Peter Dome, Petr Winkler, Raimo K. R. Salokangas, Tiina From, Vita Danileviciute, Xenia Gonda, Zoltan Rihmer, Jonas Forsman, Anne Grady, Thomas Hyphantis, Ingrid Dieset, Susan Soendergaard, Maurizio Pompili, Per Bech

**Affiliations:** 1 3rd Department of Psychiatry, School of Medicine, Aristotle University of Thessaloniki, Thessaloniki, Greece; 2 Aristotle University of Thessaloniki, Thessaloniki, Greece; 3 Department of Music Studies, School of Fine Arts, Aristotle University of Thessaloniki, Thessaloniki, Greece; 4 Department of Meteorology and Climatology, School of Geology, Aristotle University of Thessaloniki, Thessaloniki, Greece; 5 Department of Psychiatry, Psychotherapy and Psychosomatics, Center for Social Psychiatry, University Hospital of Psychiatry, Zurich, Switzerland; 6 Department of Clinical Psychology, Faculty of Psychology and Education, VU University Amsterdam, Van der Boechorststraat 1, 1081 BT Amsterdam, The Netherlands; 7 Clinic of Psychiatric, Faculty of Medicine, Vilnius University, Vilnius, Lithuania; 8 National Institute of Mental Health, Klecany, Czech Republic; 9 Institute of Mental Health, WHO Collaborating Centre, Palmoticeva 37, 11000 Belgrade, Serbia; 10 The George Washington University, School of Medicine & School of Public Health, Washington, DC USA; 11 Department of Psychiatry and Narcology, Riga Stradins University, Tvaika Str. 2, Riga, LV 1005 Latvia; 12 Department of Psychiatry, University Hospital, SNP 1, 040 66 Košice, Slovakia; 13 Department of Psychiatry, Ruhr University Bochum, LWL-University Hospital, Alexandrinenstr.1, 44791 Bochum, Germany; 14 Department of Clinical Neuroscience, Karolinska Institutet, Solna, Sweden; 15 Psychiatric Hospital Vojnik, Celjska Cesta 37, Vojnik, Slovenia; 16 National Mental Health Center and Anti-drug, Bucharest, Romania; 17 Department of Adult Psychiatry, Poznan University of Medical Sciences, Poznan, Poland; 18 Department of Psychiatry, Sainte Marguerite Hospital, 13274 Marseille, France; 19 East London NHS Trust, London, E1 4DG UK; 20 Molecular & Cellular Therapeutics, Royal College of Surgeons in Ireland, Dublin 2, Ireland; 21 University Psychiatric Hospital, Ljubljana, Slovenia; 22 University Psychiatric Center KU Leuven, Louvain, Belgium; 23 Second Psychiatric Clinic, University Hospital for Active Treatment in Neurology and Psychiatry “Sveti Naum”, Sofia, Bulgaria; 24 Psychiatric Clinic, Clinical Center of Montenegro, School of Medicine, University of Montenegro, Podgorica, Montenegro; 25 Division of Biological Psychiatry, Department of Psychiatry and Psychotherapy, Medical University of Vienna, Vienna, Austria; 26 Faculty of Medicine, University of Lisbon, Av. Prof. Egas Moniz, 1649-035 Lisbon, Portugal; 27 Department of Psychiatry, University Hospital Center Zagreb, Zagreb, Croatia; 28 Centre for Epidemiology, Surveillance and Health Promotion (CNESPS), National Institute of Health (ISS), Rome, Italy; 29 Psychiatry Unit, Department of Clinical and Experimental Medicine, University of Pisa, Pisa, Italy; 30 Institute of Behavioral Sciences “G. De Lisio”, Pisa, Italy; 31 NORMENT, KG Jebsen Centre for Psychosis Research, Institute of Clinical Medicine, University of Oslo, Oslo, Norway; 32 Division of Mental Health and Addiction, Oslo University Hospital, Oslo, Norway; 33 Institute of Mental Health, School of Medicine, University of Belgrade, Belgrade, Serbia; 34 Estonian-Swedish Mental Health and Suicidology Institute, Tallinn, Estonia; 35 Department of Clinical and Theoretical Mental Health, Faculty of Medicine, Semmelweis University, Budapest, Hungary; 36 Laboratory for Suicide Research and Prevention, National Institute of Psychiatry and Addictions, Budapest, Hungary; 37 Department of Psychiatry, University of Turku, Turku, Finland; 38 Department of Pharmacodynamics, MTA-SE, Semmelweis University, Budapest, Hungary; 39 Neuropsychopharmacology and Neurochemistry Research Group, Hungarian Academy of Sciences, Budapest, Hungary; 40 Department of Psychiatry, Ioannina School of Medicine, Ioannina, Greece; 41 Psychiatric Research Unit, Mental Health Centre North Zealand, University of Copenhagen, Dyrehavevej 48, 3400 Hillerød, Denmark; 42 Department of Neurosciences, Mental Health and Sensory Organs, Suicide Prevention Center, Sant’Andrea Hospital, Sapienza University of Rome, Rome, Italy

**Keywords:** Suicide, Europe, Austerity, Climate

## Abstract

**Background:**

It is well known that suicidal rates vary considerably among European countries and the reasons for this are unknown, although several theories have been proposed. The effect of economic variables has been extensively studied but not that of climate.

**Methods:**

Data from 29 European countries covering the years 2000–2012 and concerning male and female standardized suicidal rates (according to WHO), economic variables (according World Bank) and climate variables were gathered. The statistical analysis included cluster and principal component analysis and categorical regression.

**Results:**

The derived models explained 62.4 % of the variability of male suicidal rates. Economic variables alone explained 26.9 % and climate variables 37.6 %. For females, the respective figures were 41.7, 11.5 and 28.1 %. Male suicides correlated with high unemployment rate in the frame of high growth rate and high inflation and low GDP per capita, while female suicides correlated negatively with inflation. Both male and female suicides correlated with low temperature.

**Discussion:**

The current study reports that the climatic effect (cold climate) is stronger than the economic one, but both are present. It seems that in Europe suicidality follows the climate/temperature cline which interestingly is not from south to north but from south to north-east. This raises concerns that climate change could lead to an increase in suicide rates. The current study is essentially the first successful attempt to explain the differences across countries in Europe; however, it is an observational analysis based on aggregate data and thus there is a lack of control for confounders.

**Electronic supplementary material:**

The online version of this article (doi:10.1186/s12991-016-0106-2) contains supplementary material, which is available to authorized users.

## Background

Especially after the 2008 global economic crisis, several authors expressed concern on the effect of austerity on healthcare and especially on suicidality. It is widely believed that crises of this kind increase suicides [1–7], with men of working age being at the highest risk. There are several studies published until now, suggesting the presence of such a pattern concerning the impact of the economic crisis in European countries [6, 8–18], Asia [19, 20] and the US [15] although different and more complex interpretations also exist [21–26].

**Fig. 1:**
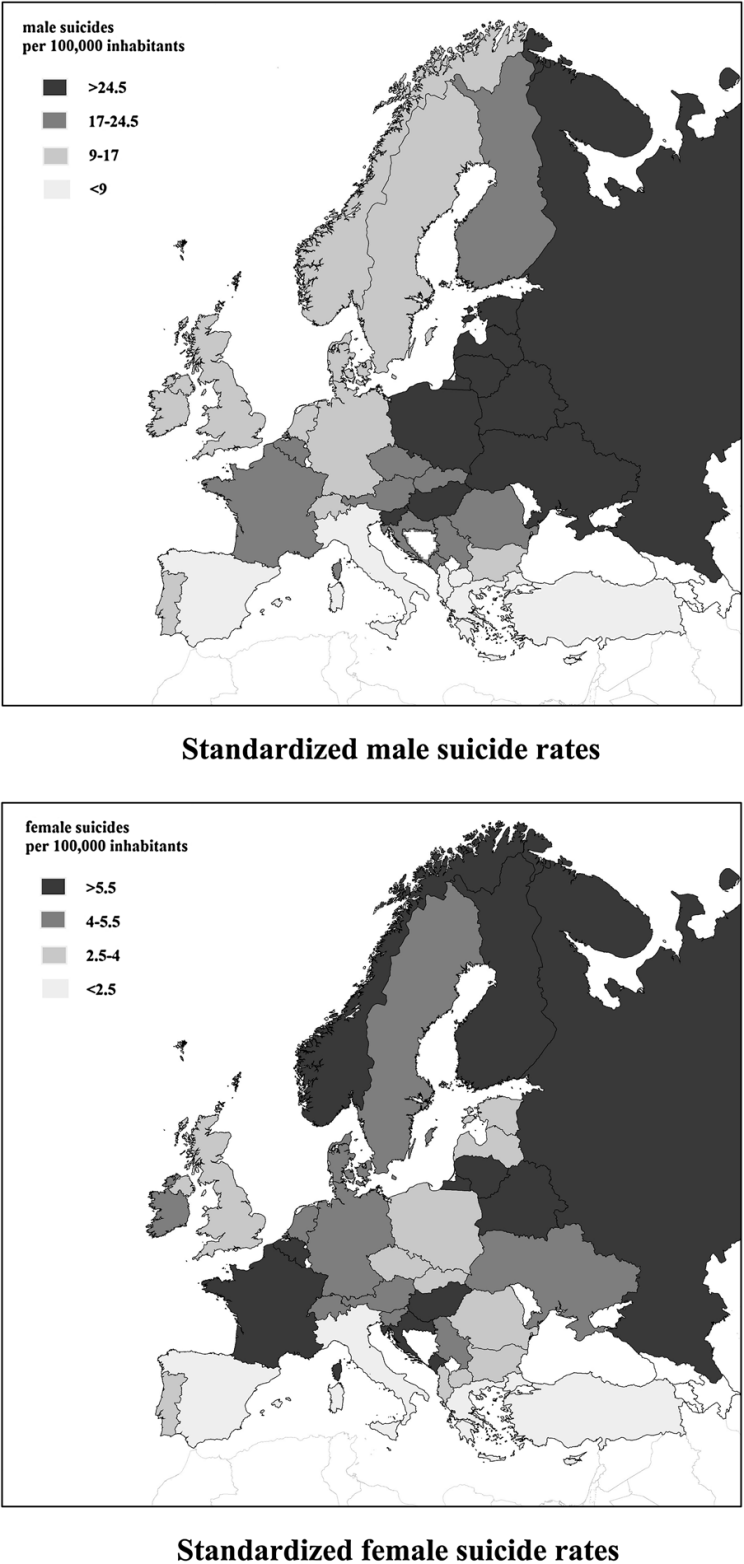
Map of male and female standardized suicide rates in Europe

Our multinational workgroup has published on the relationship of unstandardized suicidal rates with economic factors [26] and the current study constitutes an effort to investigate the effect of climate factors and their possible interplay with economic ones in Europe. It is well known that suicidal rates vary considerably among European countries (Fig. 1) and the reasons for this are unknown although several theories have been proposed. The effect of climate has previously been discussed but has not been investigated in a systematic way across countries.

## Methods

Data were gathered from 29 European countries for the years 2000–2012. They included male and female standardized suicidal rates (according to WHO), economic variables according to the World Bank (http://data.worldbank.org/) and climate variables which were calculated from the daily E-OBS gridded dataset with a spatial resolution of 0.22 degree on a rotated grid which is based on observational data.

A detail description of the methodology in the gathering of data, a list of variables used and the respected definitions are described in Additional file 1, while the entire dataset which was used in the current study is shown in Table A.

The statistical analysis included cluster analysis of variables (separately for economic and climate variables) and principal component analysis to identify prominent variables to be used afterwards in a categorical regression to test for the relationship of suicidal rates (dependent variables—DV) with economic and climate components (independent variables—IV). The method and the procedure of the statistical analyses are shown in details in Additional file 1.

## Results

For males, the regression analysis (see Additional file 1 for details) returned an *R* = 0.790 and *R-square* = 0.624 (adjusted *R*^*2*^ = 0.602) with a standard error of estimate equal to 0.376. This model explained 62.4 % of the variability of observed male suicidal rates with the combination of all the available variables. Economic variables alone could explain up to 26.9 % and climate variables alone up to 37.6 %.

For females, the results of a similar analysis returned an *R* = 0.645 and *R-square* = 0.417 (adjusted *R*^*2*^ = 0.391) with a standard error of estimate equals to 0.583. This model explained 41.7 % of the variability of observed female suicide rates with the combination of all the available variables. Economic variables alone could explain up to 11.5 % and climate variables alone up to 28.1 %. These models had very good predictive validity and fitted the data well.

The interaction of economic variables (see Additional file 1) suggests that male suicides correlate with high unemployment rate in the frame of high growth rate and high inflation and low GDP per capita, while female suicides correlate negatively with inflation. Both male and female suicides correlate with low temperature both maximum and minimum (overall cold climate).

All the detailed results can be found in the accompanying Additional file 1.

## Discussion

The current study reports that both economic and climatic variables are strongly correlated with suicide rates and can explain much of the variability observed across the European continent especially in males. The novel contribution of the current study, which is the first one to investigate these variables together in a single model, is that the climatic effect is stronger than the economic one. Together they explain 62.4 % of male and 41.7 % of female suicide rate variability across the continent.

One of the biggest enigmas is the marked geographic variability in suicide rates found in Europe, with the highest rates being found in Eastern Europe and the lowest in the Mediterranean region (Fig. 1) [26–28]. Reasons for these great differences between national/regional suicide rates have not been fully explained yet. Geographic (latitude, longitude, altitude) climatic, dietary, genetic, economic, religious and other socio-cultural differences can be taken into account, but an additional problem is that there is probably an intercorrelation between them. However, the differences in the psychiatric morbidity (including alcohol abuse), as well as the accuracy of the registration of suicide, the stigma associated with mental illness and suicide (possibly influencing help-seeking behaviour and reporting rates), the availability of lethal methods, and the availability and quality of the social/health care systems should also be considered [27, 29].

The link between economic variables and suicidality has been the focus of extensive research in the past although a clear cause and effect relationship has not been solidly established because the temporal relationship suggests that suicide rate increase precedes the increase in unemployment and other variables which indicate recession [25, 30]. In contrast, the literature on the effect of climate is limited.

While there are no reports correlating differences in suicides with differences in climate between countries and regions of the world, there is a significant literature concerning the seasonal pattern of suicides and suicide attempts. The data are complex and a number of confounding variables exist, including gender, social cues and diagnosis [31–39].

The literature suggests that overall the suicide rate is higher in autumn [40] but also during the summer with the lowest observed during winter [41–44]. Other papers report higher rates during spring and summer [45–47] or spring and autumn [48] or only spring [49–53]. There is a positive linear relation between the variation in suicide rate and geographic latitude [54], and this is true for both hemispheres but not for the tropical zone where there seems to be no seasonal pattern [55–57]. On the contrary, seasonality seems to be more pronounced in those countries closer to the poles [41] and seems to correlate with male gender and violent methods [51, 52, 58, 59] as well as with the traditional agricultural societies [50, 58]. However, some authors did not find any seasonality concerning the methods used [40].

The first ever study on the effect of climate on suicides reported that falling barometric pressure was correlated with increasing suicides [60] but this was not confirmed by latter studies. Most reports suggest that the suicide rates are higher during periods of high temperature [47–49, 51, 52, 61–73], low rainfalls [47, 63, 72, 74, 75] and more sunshine [41, 44, 45, 61, 68, 70, 76–78]. It is interesting that it seems the temperature effect is so strong that it exerts its effect on the same day concerning suicides by a violent method [51, 52] or a day after [66].

In contrast with the above, one paper reported a positive correlation of rainfalls with increasing suicidal rates [68] and another one no effect of temperature [79]. In Italy, the distribution of deaths by suicide shows a negative relationship with mean yearly temperature values, max and min, and with sun exposure indicators, and a positive, but less significant relationship with rainfall values [75]. For females, the links between temperature and suicides are less consistent than for males, and sometimes have a reverse sign, too [65]. A negative correlation for temperature has also been reported from Taiwan [76].

Although the first ever study reported that falling barometric pressure was correlated with increasing suicides [60], one more recent study confirmed this by reporting that cloudiness and atmospheric pressure were negatively correlated [47] but another study reported the opposite [73].

It has been reported that in Kazakhstan, an increase in the mean apparent temperature by 1 degree Celsius was associated with an increase in suicide counts by 2.1 % [69] and temperature variability explains more than 60 % of the total suicide variance [71]. Overall, the climatic variables explain 63 % of suicides [75]. The current study suggests that in countries with cold climate, suicidality is higher, and this should be considered in combination with the known seasonality of suicides which is not, however, part of the present paper. It seems that in Europe, suicidality follows the climate/temperature cline which interestingly is not from south to north but from south to north-east.

Although most reports suggest that sociodemographical factors are stronger predictors in comparison to climate and seasonality [64, 80], there are opposite reports [72]. Our results suggest that climatic variables could be more important factors than socio-economic ones.

Since meteorological variables seem to have an impact on mental health, there are concerns that climate change could lead to an increase in the rates of mental disorders and especially addictions and suicide rates [81]. However, this is highly unlikely to explain the high impact on suicidality from rather benign increases in temperature. It seems also that extended periods of light in the summer may contribute to impulsive–aggressive summer suicides [41], while abrupt temperature changes twice a year seem to trigger the activity in brown adipose tissue and deepen depression [48]. In this frame sunshine, via interactions with serotonin neurotransmission, may trigger increased impulsivity and promote suicidal acts [78].

The current study is the first successful attempt to explain the large differences between European countries in terms of suicidal rates. It also suggests the presence of different underlying mechanisms for males and females pertaining to the interaction with different qualities of environmental stimuli.

However, it suffers from a number of limitations. It is an observational analysis based on aggregate data collected from national statistical agencies. Thus, there is a lack of ability to deeper investigation and understanding of the structure. Probably, there are differences between countries both in the quality of the data as well as in the level of misclassification of suicide, and these could lead to potential bias between countries [82], but it is not expected they had a significant impact on the results of the current study.

Cross-level bias and aggregation bias are typical of studies similar to the current one [83]. The effects observed on the aggregate level might be modulated by the ecological context at the level of the individual person [84]. Also time series data are frequently non-stationary and vulnerable to random findings [84]. Finally, another source of bias is the possible registration bias concerning suicides between countries and over time, and also concerning the quality of the economical statistics.

The authors chose to publish the full database their analysis was based on in an appendix, since they strongly believe that this database should be publicly available, so that anyone could perform further analysis which is one of the major contributions of the current study.

## Conclusions

The current study reports that both economic and climatic variables are strongly correlated with suicide rates and can explain much of the variability observed across the European continent especially in males. However, the climatic effect is stronger than the economic one concerning both sexes, but the relative effect of climate in comparison to economic variables was higher for females (ratio climate to economy effect: 2.44 in females vs. 1.39 in males). Together they explain 62.4 % of male and 41.7 % of female suicide rate variability across the continent.

## Additional file

10.1186/s12991-016-0106-2 Web appendix.
