# Supplementary material for: Relationship of suicide rates with climate and economic variables in Europe during 2000–2012
Source: Ann Gen Psychiatry. 2016 Aug 9;15:19. doi: 10.1186/s12991-016-0106-2 (PMC4977895; doi:10.1186/s12991-016-0106-2)
Supplement: Supplementary file 1 — 10.1186/s12991-016-0106-2 Web appendix. [file 12991_2016_106_MOESM1_ESM.docx]

**Webappendix**

to Fountoulakis et al: Relationship of suicide rates with climate and economic variables in Europe during 2000-2012

**Contents**

*1. Background*

*2. Data sources*

*3. Material*

*4. Statistical analysis*

*5 Results*

*5a. Cluster analysis (CA)*

*5b Principal Components Analysis (PCA)*

*5c Categorical Regression (CATREG) and Linear Multiple Regression (LMR)*

*5c-1 Males*

*5c-1a Model M1m (all IV untransformed, logarithmic transformation of DV)*

*5c-1b Model M2m (all IV optimaly transformed, DV untransformed)*

*5c-1c Model Mme (only economic variables as IV)*

*5c-1d Model Mmc (only climate variables as IV)*

*5c-2 Females*

*5c-2a Model M1f (all IV untransformed, logarithmic transformation of DV)*

*5c-2b Model M2f (all IV optimaly transformed, DV untransformed)*

*5c-2c Model Mfe (only economic variables as IV)*

*5c-2d Model Mfc (only climate variables as IV)*

*6. Summary of results*

*Note:* IV: independent variables

DV: dependent variable

1. **Background**

The current paper investigates the possible relation between suicidal rates, climate and economic variables. The main idea was to identify the components of economic and climate quantities that emerge as independent factors and afterwards to explore their relation to suicidal rates. It is important to note that the methodology concerned an overall multiple regression rather than a time-series modeling.

1. **Data sources:**

The standardized suicide rates were obtained from the WHO website (<http://apps.who.int/healthinfo/statistics/mortality/whodpms/>). The economic variables were obtained from the World Bank (<http://data.worldbank.org/>) while the climate variables were calculated from the daily E-OBS gridded dataset with a spatial resolution of 0.22 deg on a rotated grid which is based on observational data.

1. **Material**

The complete dataset concerning 29 European countries on which the current analysis relied is shown in webappendix table A.

| **Year** | **Male suicides** | **Female suicides** | **National unemployment rate** | **National growth**  **rate** | **GDP per**  **capita in USD** | **inflation** | **Rainfall** | **Temperature** | **Monthly**  **Max Temperature** | **Monthly**  **Min Temperature** | **Temperature Range**  **anual** |
| --- | --- | --- | --- | --- | --- | --- | --- | --- | --- | --- | --- |
| **Austria** | | | | | | | | | | | |
| 2000 | 25.10 | 7.80 | 3.60 | 3.67 | 24517.30 | 2.00 | 37.94 | 8.03 | 17.35 | -3.88 | 21.23 |
| 2001 | 23.40 | 7.10 | 3.60 | 0.86 | 24489.70 | 2.30 | 33.97 | 6.85 | 17.33 | -4.86 | 22.19 |
| 2002 | 25.20 | 6.40 | 4.20 | 1.69 | 26351.40 | 1.70 | 39.47 | 7.74 | 16.75 | -2.37 | 19.12 |
| 2003 | 22.30 | 6.90 | 4.30 | 0.87 | 32102.90 | 1.30 | 29.17 | 7.45 | 19.49 | -4.55 | 24.04 |
| 2004 | 22.40 | 6.00 | 4.90 | 2.59 | 36695.00 | 2.00 | 36.39 | 6.77 | 16.42 | -4.04 | 20.46 |
| 2005 | 21.10 | 6.00 | 5.20 | 2.40 | 38241.10 | 2.10 | 34.98 | 6.50 | 16.27 | -4.59 | 20.87 |
| 2006 | 19.80 | 4.80 | 4.80 | 3.67 | 40428.90 | 1.70 | 32.32 | 7.23 | 19.26 | -5.33 | 24.59 |
| 2007 | 19.20 | 5.10 | 4.40 | 3.71 | 46584.30 | 2.20 | 35.44 | 7.83 | 16.82 | -2.16 | 18.98 |
| 2008 | 18.10 | 5.00 | 3.80 | 1.40 | 51388.30 | 3.22 | 35.47 | 7.62 | 16.14 | -0.92 | 17.05 |
| 2009 | 18.50 | 4.90 | 4.80 | -3.81 | 47651.30 | 0.40 | 40.64 | 7.41 | 17.44 | -3.96 | 21.40 |
| 2010 | 18.50 | 4.90 | 4.40 | 1.80 | 46590.60 | 1.70 | 35.92 | 6.42 | 18.14 | -4.62 | 22.77 |
| 2011 | 18.10 | 5.30 | 4.20 | 2.80 | 51134.10 | 3.60 | 29.24 | 7.78 | 17.35 | -2.25 | 19.61 |
| 2012 |  |  |  |  | 48348.20 |  | 37.52 | 7.49 | 17.53 | -5.40 | 22.93 |
| **Belgium** | | | | | | | | | | | |
| 2000 |  |  |  |  | 23151.90 |  | 32.09 | 10.50 | 17.65 | 3.05 | 14.60 |
| 2001 |  |  |  |  | 23078.40 |  | 33.70 | 9.97 | 18.63 | 1.68 | 16.94 |
| 2002 |  |  |  |  | 25006.80 |  | 33.37 | 10.55 | 18.14 | 3.48 | 14.66 |
| 2003 | 25.90 | 8.70 | 8.2 | 0.8 | 30702.50 | 1.59 | 21.92 | 10.32 | 19.94 | 1.39 | 18.55 |
| 2004 | 24.10 | 8.20 | 8.4 | 3.3 | 35549.00 | 2.1 | 27.27 | 9.76 | 18.60 | 2.02 | 16.58 |
| 2005 | 23.90 | 8.20 | 8.5 | 1.8 | 36927.10 | 2.78 | 24.54 | 10.18 | 17.91 | 0.97 | 16.95 |
| 2006 | 22.70 | 8.00 | 8.3 | 2.7 | 38934.40 | 1.79 | 28.31 | 10.72 | 22.34 | 0.65 | 21.69 |
| 2007 | 21.10 | 7.60 | 7.5 | 2.9 | 44447.40 | 1.82 | 30.93 | 11.09 | 17.30 | 3.65 | 13.65 |
| 2008 | 23.00 | 8.10 | 7 | 1 | 48563.10 | 4.49 | 28.08 | 10.37 | 17.66 | 2.26 | 15.40 |
| 2009 | 22.80 | 8.20 | 7.9 | -2.8 | 44996.50 | -0.04 | 26.54 | 10.52 | 18.96 | 0.14 | 18.82 |
| 2010 | 22.60 | 8.20 | 8.3 | 2.4 | 44358.30 | 2.19 | 26.75 | 9.20 | 20.17 | -1.41 | 21.58 |
| 2011 |  |  |  |  | 47814.10 |  | 23.00 | 11.20 | 17.40 | 3.37 | 14.02 |
| 2012 |  |  |  |  | 44827.70 |  | 29.27 | 10.26 | 18.91 | -0.09 | 19.00 |
| **Bulgaria** | | | | | | | | | | | |
| 2000 | 20.50 | 6.40 |  |  | 1634.40 |  | 9.10 | 11.65 | 23.58 | -3.59 | 27.18 |
| 2001 | 19.40 | 6.10 |  |  | 1783.50 |  | 15.04 | 11.42 | 23.30 | -3.90 | 27.21 |
| 2002 | 20.00 | 5.60 |  |  | 2077.10 |  | 20.21 | 11.36 | 22.98 | -0.61 | 23.59 |
| 2003 | 16.40 | 5.00 |  |  | 2697.20 |  | 16.31 | 10.63 | 23.39 | -3.65 | 27.04 |
| 2004 | 15.20 | 4.40 |  |  | 3331.10 |  | 16.74 | 10.90 | 21.15 | -1.74 | 22.88 |
| 2005 | 14.60 | 4.40 | 10.20 | 6.40 | 3785.70 | 5.00 | 25.75 | 10.50 | 21.24 | -0.77 | 22.01 |
| 2006 | 15.20 | 3.70 | 9.00 | 6.60 | 4370.60 | 7.30 | 17.34 | 11.34 | 22.55 | -2.02 | 24.57 |
| 2007 | 13.00 | 4.00 | 6.90 | 6.40 | 5783.00 | 8.40 | 22.32 | 12.50 | 24.65 | 0.45 | 24.20 |
| 2008 | 14.10 | 3.90 | 5.70 | 6.20 | 7115.90 | 12.30 | 13.91 | 12.18 | 23.73 | -1.21 | 24.93 |
| 2009 | 13.20 | 3.30 | 6.90 | -5.50 | 6738.10 | 2.80 | 20.06 | 12.10 | 22.74 | 0.88 | 21.85 |
| 2010 | 13.10 | 3.40 | 10.30 | 0.40 | 6580.80 | 2.40 | 22.75 | 12.11 | 24.39 | -0.11 | 24.50 |
| 2011 | 12.70 | 2.70 |  | 2 | 7588.80 | 3.4 | 14.42 | 11.32 | 22.81 | 0.56 | 22.25 |
| 2012 | 13.90 | 3.50 |  | 0.5 | 7198.00 | 2.4 | 18.17 | 12.33 | 25.50 | -3.01 | 28.50 |
| **Croatia** | | | | | | | | | | | |
| 2000 | 30.40 | 7.60 | 22.60 | 2.90 | 4919.60 | 5.50 | 26.81 | 12.30 | 22.53 | -0.96 | 23.49 |
| 2001 | 25.80 | 6.70 | 15.30 | 4.40 | 5245.40 | 2.40 | 33.22 | 11.35 | 22.33 | -1.91 | 24.23 |
| 2002 | 24.90 | 6.90 | 15.20 | 5.20 | 6053.70 | 1.80 | 34.36 | 12.14 | 21.64 | 0.67 | 20.96 |
| 2003 | 25.70 | 5.60 | 14.10 | 5.40 | 7805.90 | 1.70 | 22.55 | 11.50 | 24.20 | -1.86 | 26.06 |
| 2004 | 23.90 | 7.10 | 13.80 | 4.10 | 9365.70 | 2.70 | 35.07 | 10.80 | 20.88 | -0.47 | 21.36 |
| 2005 | 23.90 | 6.80 | 12.70 | 4.30 | 10224.30 | 3.60 | 35.84 | 10.25 | 20.94 | -1.70 | 22.64 |
| 2006 | 21.50 | 6.50 | 11.10 | 4.90 | 11359.50 | 2.10 | 28.36 | 11.26 | 22.78 | -1.06 | 23.84 |
| 2007 | 21.10 | 5.70 | 9.60 | 5.10 | 13540.40 | 5.80 | 29.60 | 12.07 | 22.47 | 0.67 | 21.80 |
| 2008 | 22.00 | 5.30 | 8.40 | 2.10 | 15889.40 | 2.90 | 30.24 | 12.00 | 21.41 | 3.04 | 18.37 |
| 2009 | 22.50 | 4.50 | 9.10 | -6.90 | 14142.20 | 1.90 | 32.37 | 12.04 | 22.26 | -0.30 | 22.56 |
| 2010 | 20.80 | 5.60 | 11.80 | -1.40 | 13500.90 | 1.80 | 42.82 | 11.06 | 22.50 | -0.20 | 22.70 |
| 2011 | 18.50 | 5.30 | 17.80 | 0.00 | 14540.30 | 1.20 | 19.92 | 11.84 | 22.61 | 1.59 | 21.02 |
| 2012 | 21.90 | 5.20 |  |  | 13234.60 |  | 30.69 | 12.26 | 23.62 | -2.54 | 26.15 |
| **Czech Republic** | | | | | | | | | | | |
| 2000 | 22.40 | 5.00 | 8.78 | 4.20 | 5994.50 | 3.90 | 19.95 | 9.06 | 18.34 | -2.26 | 20.61 |
| 2001 | 22.10 | 4.70 | 8.90 | 3.10 | 6594.70 | 4.70 | 23.36 | 7.72 | 18.10 | -3.43 | 21.54 |
| 2002 | 20.60 | 4.70 | 9.81 | 2.10 | 8011.90 | 1.80 | 25.40 | 8.59 | 18.46 | -2.80 | 21.26 |
| 2003 | 23.30 | 5.00 | 10.31 | 3.80 | 9741.10 | 0.10 | 14.49 | 8.29 | 20.23 | -4.08 | 24.32 |
| 2004 | 21.70 | 4.10 | 9.47 | 4.70 | 11667.70 | 2.80 | 19.41 | 7.84 | 18.11 | -3.72 | 21.82 |
| 2005 | 21.20 | 4.20 | 8.88 | 6.80 | 13317.70 | 1.90 | 20.38 | 7.66 | 17.79 | -3.47 | 21.25 |
| 2006 | 18.80 | 3.60 | 7.67 | 7.00 | 15159.20 | 2.50 | 19.81 | 8.22 | 21.31 | -5.79 | 27.10 |
| 2007 | 18.70 | 3.20 | 5.98 | 5.70 | 18334.00 | 2.80 | 21.86 | 8.97 | 18.10 | -0.76 | 18.86 |
| 2008 | 18.10 | 3.70 | 5.96 | 3.10 | 22649.40 | 6.30 | 17.69 | 8.79 | 17.83 | 0.60 | 17.23 |
| 2009 | 19.30 | 3.30 | 9.24 | -4.70 | 19698.50 | 1.00 | 21.25 | 8.38 | 18.44 | -3.95 | 22.39 |
| 2010 | 19.60 | 3.70 | 9.57 | 2.70 | 19764.00 | 1.50 | 25.01 | 7.19 | 19.91 | -4.88 | 24.79 |
| 2011 | 21.10 | 3.70 |  |  | 21656.40 |  | 18.63 | 8.73 | 18.12 | -1.80 | 19.92 |
| 2012 | 21.40 | 3.70 |  |  | 19670.40 |  | 20.64 | 8.49 | 18.52 | -5.06 | 23.58 |
| **Denmark** | | | | | | | | | | | |
| 2000 | 16.80 | 5.40 | 4.60 | 3.50 | 30743.60 | 2.90 | 22.87 | 10.02 | 16.82 | 3.08 | 13.75 |
| 2001 | 16.00 | 5.80 | 4.80 | 0.70 | 30751.60 | 2.40 | 22.52 | 9.15 | 18.94 | 0.84 | 18.10 |
| 2002 | 15.80 | 4.90 | 4.80 | 0.50 | 33228.70 | 2.40 | 26.67 | 9.91 | 21.08 | 0.38 | 20.70 |
| 2003 | 14.70 | 4.30 | 5.60 | 0.40 | 40458.80 | 2.10 | 19.07 | 9.27 | 19.32 | -0.73 | 20.05 |
| 2004 | 14.70 | 5.70 | 5.70 | 2.30 | 46487.80 | 1.20 | 24.42 | 9.04 | 18.54 | -0.18 | 18.72 |
| 2005 | 13.70 | 4.60 | 5.00 | 2.40 | 48816.80 | 1.80 | 19.38 | 9.20 | 17.77 | 0.73 | 17.04 |
| 2006 | 14.00 | 5.00 | 4.10 | 3.40 | 52041.20 | 1.90 | 24.58 | 9.71 | 20.19 | -0.47 | 20.66 |
| 2007 | 12.10 | 4.90 | 3.80 | 1.60 | 58501.10 | 1.70 | 25.75 | 9.81 | 17.21 | 2.29 | 14.92 |
| 2008 | 12.60 | 5.10 | 3.50 | -0.80 | 64181.60 | 3.40 | 23.07 | 9.97 | 17.98 | 3.46 | 14.52 |
| 2009 | 13.90 | 3.70 | 6.10 | -5.70 | 57895.90 | 1.30 | 21.94 | 9.32 | 17.95 | 1.16 | 16.79 |
| 2010 | 11.70 | 4.20 | 7.60 | 1.60 | 57647.90 | 2.30 | 21.89 | 7.42 | 18.87 | -2.61 | 21.47 |
| 2011 | 12.70 | 4.10 | 7.70 | 1.10 | 61303.90 | 2.80 | 24.05 | 9.18 | 16.64 | 0.06 | 16.57 |
| 2012 |  |  | 6.1 | -0.6 | 57636.10 | 2.4 | 24.38 | 8.70 | 17.42 | -0.36 | 17.77 |
| **Estonia** | | | | | | | | | | | |
| 2000 | 41.40 | 9.10 | 13.60 | 9.70 | 4069.90 | 4.00 | 21.90 | 7.10 | 16.48 | -2.65 | 19.13 |
| 2001 | 45.40 | 8.40 | 12.60 | 6.30 | 4490.60 | 5.80 | 27.23 | 6.06 | 20.62 | -7.53 | 28.15 |
| 2002 | 43.10 | 7.40 | 10.30 | 6.60 | 5298.60 | 3.60 | 18.14 | 6.25 | 19.17 | -8.52 | 27.69 |
| 2003 | 39.10 | 6.70 | 10.00 | 7.80 | 7165.70 | 1.30 | 21.07 | 5.77 | 19.89 | -7.39 | 27.27 |
| 2004 | 37.60 | 6.30 | 9.70 | 6.30 | 8849.30 | 3.00 | 23.36 | 6.01 | 17.48 | -7.18 | 24.66 |
| 2005 | 30.90 | 4.90 | 7.90 | 8.90 | 10336.10 | 4.10 | 13.59 | 5.92 | 18.19 | -6.66 | 24.85 |
| 2006 | 26.10 | 5.00 | 5.90 | 10.10 | 12591.30 | 4.40 | 13.70 | 6.71 | 18.97 | -8.60 | 27.57 |
| 2007 | 28.60 | 4.10 | 4.70 | 7.50 | 16580.10 | 6.60 | 19.62 | 7.10 | 18.58 | -9.29 | 27.87 |
| 2008 | 26.40 | 5.60 | 5.50 | -4.20 | 18088.40 | 10.40 | 24.38 | 7.42 | 16.75 | -0.73 | 17.48 |
| 2009 | 29.90 | 4.40 | 13.80 | -14.10 | 14717.40 | -0.10 | 22.76 | 6.16 | 17.41 | -4.51 | 21.92 |
| 2010 | 24.70 | 4.00 | 16.90 | 3.30 | 14629.60 | 3.00 | 21.87 | 5.27 | 22.37 | -12.80 | 35.17 |
| 2011 | 23.70 | 4.10 | 12.50 | 8.30 | 17178.50 | 5.00 | 18.10 | 7.09 | 20.94 | -10.19 | 31.13 |
| 2012 | 25.30 | 3.90 |  |  | 17132.20 |  | 24.49 | 5.63 | 18.61 | -9.55 | 28.16 |
| **Finland** | | | | | | | | | | | |
| 2000 | 31.00 | 9.70 | 9.80 | 5.30 | 24253.30 | 3.40 | 20.91 | 3.29 | 15.69 | -7.86 | 23.54 |
| 2001 | 32.70 | 8.70 | 9.10 | 2.30 | 24913.20 | 2.60 | 18.75 | 1.74 | 16.71 | -12.15 | 28.85 |
| 2002 | 28.80 | 8.70 | 9.10 | 1.80 | 26834.00 | 1.60 | 16.28 | 1.89 | 16.88 | -12.89 | 29.77 |
| 2003 | 28.30 | 8.40 | 9.00 | 2.00 | 32816.20 | 0.90 | 17.25 | 2.24 | 18.71 | -16.18 | 34.89 |
| 2004 | 28.10 | 8.50 | 8.80 | 4.10 | 37637.70 | 0.20 | 21.05 | 2.37 | 15.93 | -10.15 | 26.09 |
| 2005 | 24.00 | 8.90 | 8.40 | 2.90 | 38968.20 | 0.90 | 20.21 | 3.05 | 17.02 | -8.80 | 25.81 |
| 2006 | 27.30 | 8.00 | 7.70 | 4.40 | 41118.60 | 1.60 | 16.89 | 2.66 | 15.86 | -12.55 | 28.41 |
| 2007 | 25.10 | 7.80 | 6.90 | 5.30 | 48286.10 | 2.50 | 20.89 | 2.84 | 15.03 | -15.54 | 30.57 |
| 2008 | 26.70 | 7.70 | 6.40 | 0.30 | 53403.30 | 4.10 | 22.43 | 2.92 | 14.29 | -5.45 | 19.74 |
| 2009 | 25.40 | 8.90 | 8.20 | -8.50 | 47104.30 | 0.00 | 16.03 | 2.18 | 14.65 | -9.81 | 24.45 |
| 2010 | 24.30 | 7.60 | 8.40 | 3.30 | 46202.40 | 1.20 | 17.74 | 0.91 | 18.38 | -14.90 | 33.28 |
| 2011 | 23.30 | 6.60 | 7.80 | 2.70 | 50790.70 | 3.40 | 21.08 | 3.35 | 17.56 | -16.06 | 33.61 |
| 2012 |  |  |  |  | 47243.70 |  | 23.16 | 1.59 | 15.27 | -12.47 | 27.74 |
| **France** | | | | | | | | | | | |
| 2000 | 23.40 | 7.50 | 8.50 | 3.70 | 22466.20 | 1.70 | 30.90 | 11.72 | 19.72 | 3.35 | 16.37 |
| 2001 | 22.40 | 7.30 | 7.70 | 1.80 | 22527.90 | 1.70 | 29.14 | 11.32 | 20.00 | 2.26 | 17.75 |
| 2002 | 22.40 | 7.50 | 7.90 | 0.90 | 24276.00 | 1.90 | 29.32 | 11.80 | 18.35 | 4.92 | 13.43 |
| 2003 | 23.00 | 7.30 | 8.50 | 0.90 | 29692.30 | 2.10 | 22.67 | 12.10 | 23.44 | 2.56 | 20.88 |
| 2004 | 22.20 | 7.50 | 8.90 | 2.50 | 33877.70 | 2.10 | 24.53 | 11.19 | 19.48 | 3.79 | 15.69 |
| 2005 | 21.70 | 7.20 | 8.90 | 1.80 | 34880.60 | 1.80 | 19.64 | 11.28 | 19.76 | 2.10 | 17.66 |
| 2006 | 20.90 | 7.00 | 8.80 | 2.50 | 36544.60 | 1.60 | 23.39 | 12.02 | 23.20 | 2.68 | 20.53 |
| 2007 | 20.20 | 6.60 | 8.00 | 2.30 | 41600.80 | 1.50 | 25.22 | 11.74 | 18.33 | 3.96 | 14.37 |
| 2008 | 20.60 | 6.60 | 7.40 | -0.10 | 45417.50 | 2.80 | 27.80 | 11.36 | 19.02 | 3.31 | 15.70 |
| 2009 | 20.80 | 6.50 | 9.10 | -2.70 | 41631.30 | 0.10 | 22.61 | 11.77 | 20.72 | 1.96 | 18.76 |
| 2010 | 20.30 | 6.50 | 9.40 | 1.50 | 40706.10 | 1.50 | 24.72 | 10.60 | 20.89 | 1.16 | 19.73 |
| 2011 |  |  | 9.60 | 1.70 | 43809.70 | 2.10 | 21.29 | 12.44 | 19.50 | 3.89 | 15.61 |
| 2012 |  |  | 10.6 | 0.3 | 40925.20 | 1.17 | 25.98 | 11.52 | 20.68 | 0.53 | 20.16 |
| **Germany** | | | | | | | | | | | |
| 2000 | 16.50 | 4.90 | 7.40 | 3.20 | 23685.40 | 1.40 | 25.12 | 9.90 | 17.60 | 1.09 | 16.51 |
| 2001 | 16.50 | 4.80 | 7.50 | 1.20 | 23654.30 | 1.90 | 28.75 | 9.04 | 18.62 | -0.20 | 18.82 |
| 2002 | 16.10 | 5.00 | 8.30 | 0.00 | 25170.80 | 1.50 | 31.79 | 9.56 | 18.84 | 0.27 | 18.56 |
| 2003 | 16.10 | 4.90 | 9.20 | -0.20 | 30318.50 | 1.00 | 18.63 | 9.39 | 20.55 | -1.87 | 22.42 |
| 2004 | 15.40 | 4.40 | 9.70 | 1.20 | 34121.70 | 1.70 | 24.75 | 8.96 | 18.46 | -0.07 | 18.53 |
| 2005 | 14.50 | 4.40 | 10.60 | 0.80 | 34649.90 | 1.50 | 23.54 | 9.02 | 18.05 | -0.99 | 19.03 |
| 2006 | 13.60 | 4.10 | 9.80 | 3.40 | 36399.60 | 1.60 | 22.70 | 9.59 | 22.04 | -2.58 | 24.62 |
| 2007 | 13.10 | 3.80 | 8.30 | 2.70 | 41760.80 | 2.30 | 29.78 | 9.88 | 17.41 | 1.63 | 15.79 |
| 2008 | 13.10 | 3.90 | 7.20 | 1.00 | 45634.50 | 2.60 | 23.57 | 9.51 | 18.04 | 1.06 | 16.98 |
| 2009 | 13.30 | 3.90 | 7.40 | -4.70 | 41668.80 | 0.40 | 24.30 | 9.20 | 18.67 | -2.15 | 20.83 |
| 2010 | 13.80 | 4.10 | 6.80 | 3.60 | 41723.40 | 1.10 | 26.11 | 7.86 | 20.37 | -3.68 | 24.05 |
| 2011 | 13.80 | 4.00 | 5.70 | 3.00 | 45870.60 | 2.30 | 22.53 | 9.66 | 17.74 | 0.95 | 16.78 |
| 2012 | 13.00 | 4.20 |  |  | 43931.70 |  | 23.59 | 9.11 | 18.44 | -2.47 | 20.90 |
| **Greece** | | | | | | | | | | | |
| 2000 | 4.60 | 1.20 | 11.35 | 3.80 | 11960.70 | 3.20 | 11.58 | 14.44 | 25.14 | 1.62 | 23.52 |
| 2001 | 4.40 | 0.70 | 10.78 | 3.60 | 12418.70 | 3.40 | 15.02 | 14.97 | 25.71 | 1.74 | 23.97 |
| 2002 | 3.80 | 0.90 | 10.31 | 3.50 | 13903.70 | 3.60 | 22.90 | 14.45 | 25.20 | 3.07 | 22.13 |
| 2003 | 4.60 | 1.00 | 9.71 | 4.70 | 18292.00 | 3.50 | 16.98 | 14.16 | 25.22 | 1.65 | 23.57 |
| 2004 | 4.20 | 0.90 | 10.49 | 3.70 | 21677.40 | 2.90 | 14.09 | 14.16 | 24.57 | 3.64 | 20.92 |
| 2005 | 4.50 | 1.20 | 9.85 | 3.70 | 22326.50 | 3.50 | 16.04 | 13.76 | 24.27 | 4.17 | 20.10 |
| 2006 | 4.60 | 1.00 | 8.89 | 4.20 | 24556.70 | 3.20 | 13.84 | 13.85 | 24.63 | 2.95 | 21.68 |
| 2007 | 3.90 | 0.90 | 8.28 | 4.00 | 28546.80 | 2.90 | 16.11 | 14.79 | 25.54 | 5.12 | 20.41 |
| 2008 | 4.30 | 0.90 | 7.65 | 2.90 | 31701.70 | 4.20 | 11.69 | 14.86 | 24.94 | 4.80 | 20.13 |
| 2009 | 4.70 | 0.80 | 9.46 | -2.00 | 29483.70 | 1.21 | 16.19 | 14.58 | 24.45 | 5.58 | 18.87 |
| 2010 | 4.60 | 0.60 | 12.53 | -4.50 | 26861.50 | 4.71 | 16.60 | 15.06 | 25.77 | 6.33 | 19.44 |
| 2011 | 5.50 | 1.30 | 17.65 | -7.10 | 25964.00 | 3.33 | 13.82 | 13.94 | 24.55 | 5.83 | 18.72 |
| 2012 |  |  |  |  | 22494.40 |  | 17.92 | 14.71 | 26.31 | 2.60 | 23.70 |
| **Hungary** | | | | | | | | | | | |
| 2000 | 44.00 | 10.70 | 6.40 | 4.20 | 4613.70 | 9.80 | 11.91 | 11.89 | 22.58 | -2.33 | 24.91 |
| 2001 | 39.60 | 9.20 | 5.70 | 3.70 | 5254.80 | 9.20 | 15.14 | 10.77 | 22.29 | -4.45 | 26.74 |
| 2002 | 38.00 | 8.80 | 5.80 | 4.50 | 6631.40 | 5.30 | 14.69 | 11.68 | 23.06 | -0.87 | 23.93 |
| 2003 | 36.90 | 8.20 | 5.90 | 3.90 | 8365.50 | 4.70 | 12.40 | 10.74 | 23.66 | -4.29 | 27.94 |
| 2004 | 36.00 | 8.70 | 6.10 | 4.80 | 10206.30 | 6.80 | 19.70 | 10.48 | 20.97 | -2.24 | 23.21 |
| 2005 | 34.50 | 7.80 | 7.20 | 4.00 | 11092.40 | 3.60 | 20.96 | 10.15 | 21.14 | -2.59 | 23.73 |
| 2006 | 31.40 | 8.00 | 7.50 | 3.90 | 11342.90 | 3.90 | 16.42 | 10.90 | 23.46 | -2.53 | 25.99 |
| 2007 | 31.60 | 7.10 | 7.40 | 0.10 | 13781.10 | 8.00 | 17.23 | 12.03 | 23.21 | -0.50 | 23.71 |
| 2008 | 31.80 | 7.30 | 7.80 | 0.90 | 15598.30 | 6.10 | 17.74 | 11.80 | 21.42 | 1.35 | 20.08 |
| 2009 | 32.10 | 7.60 | 10.00 | -6.80 | 12906.80 | 4.20 | 17.05 | 11.66 | 22.54 | -1.52 | 24.06 |
| 2010 | 32.10 | 7.20 | 11.20 | 1.30 | 12958.50 | 4.90 | 27.75 | 10.71 | 22.88 | -1.55 | 24.43 |
| 2011 | 30.70 | 7.40 | 10.90 | 1.60 | 13983.50 | 3.90 | 11.45 | 11.25 | 22.28 | -0.21 | 22.49 |
| 2012 | 29.50 | 6.90 |  |  | 12784.30 |  | 13.97 | 11.74 | 23.72 | -3.61 | 27.33 |
| **Ireland** | | | | | | | | | | | |
| 2000 | 19.60 | 4.10 | 4.3 | 11.0 | 26100.70 | 5.6 | 44.85 | 9.73 | 15.38 | 5.26 | 10.12 |
| 2001 | 20.50 | 3.90 | 3.9 | 2.7 | 28051.80 | 4.9 | 31.21 | 9.64 | 14.98 | 3.79 | 11.20 |
| 2002 | 18.00 | 3.90 | 4.4 | 1.8 | 32354.30 | 4.6 | 43.84 | 10.02 | 15.24 | 5.91 | 9.33 |
| 2003 | 17.10 | 4.50 | 4.6 | 4.7 | 40904.60 | 3.5 | 30.56 | 10.14 | 16.43 | 5.04 | 11.39 |
| 2004 | 18.40 | 3.40 | 4.5 | 3.8 | 47427.70 | 2.2 | 34.76 | 10.02 | 15.63 | 5.42 | 10.21 |
| 2005 | 16.70 | 4.00 | 4.4 | 6.0 | 50567.80 | 2.5 | 33.41 | 10.18 | 15.69 | 5.03 | 10.66 |
| 2006 | 16.50 | 3.60 | 4.5 | 6.5 | 53941.30 | 4.0 | 41.41 | 10.26 | 17.05 | 5.22 | 11.84 |
| 2007 | 15.60 | 4.20 | 4.7 | 3.6 | 61215.50 | 4.9 | 39.12 | 10.38 | 14.72 | 5.71 | 9.01 |
| 2008 | 16.50 | 5.20 | 6.4 | -1.8 | 60971.10 | 4.1 | 46.43 | 9.61 | 15.00 | 4.61 | 10.39 |
| 2009 | 18.10 | 4.50 | 12.0 | -9.1 | 51493.50 | -4.5 | 48.50 | 9.58 | 14.76 | 3.06 | 11.70 |
| 2010 | 16.80 | 4.20 | 13.8 | 0.5 | 47900.80 | -1.0 | 30.89 | 8.66 | 15.26 | 0.75 | 14.52 |
| 2011 |  |  | 14.6 | -1.6 | 51951.60 | 2.6 | 34.74 | 9.99 | 14.04 | 3.65 | 10.39 |
| 2012 |  |  |  |  | 48391.30 |  | 35.49 | 9.77 | 15.62 | 5.77 | 9.85 |
| **Italy** | | | | | | | | | | | |
| 2000 | 8.60 | 2.60 | 10.40 | 3.7 | 20059.20 | 2.50 | 24.92 | 13.11 | 22.72 | 3.29 | 19.43 |
| 2001 | 8.50 | 2.40 | 9.70 | 1.8 | 20409.00 | 2.80 | 20.14 | 13.02 | 23.03 | 3.15 | 19.88 |
| 2002 | 8.70 | 2.30 | 9.10 | 0.5 | 22205.80 | 2.50 | 27.28 | 13.12 | 21.47 | 3.69 | 17.78 |
| 2003 | 8.40 | 2.40 | 8.60 | -0.05 | 27399.10 | 2.70 | 20.51 | 13.42 | 25.24 | 2.29 | 22.95 |
| 2004 |  |  |  |  | 31190.00 |  | 26.68 | 12.77 | 22.17 | 3.54 | 18.63 |
| 2005 |  |  |  |  | 31973.10 |  | 25.43 | 12.24 | 22.22 | 2.39 | 19.83 |
| 2006 | 7.30 | 2.10 | 6.79 | 2.20 | 33424.50 | 2.10 | 20.37 | 13.14 | 23.46 | 2.97 | 20.49 |
| 2007 | 7.50 | 2.00 | 6.09 | 1.68 | 37714.50 | 1.80 | 21.74 | 13.43 | 22.59 | 4.72 | 17.87 |
| 2008 | 7.70 | 2.10 | 6.74 | -1.16 | 40661.20 | 3.30 | 30.91 | 13.17 | 22.65 | 5.22 | 17.43 |
| 2009 | 7.80 | 2.10 | 7.79 | -5.49 | 36992.90 | 0.80 | 26.33 | 13.18 | 23.57 | 4.15 | 19.42 |
| 2010 | 7.90 | 2.00 |  |  | 35875.70 |  | 31.40 | 12.45 | 23.18 | 3.37 | 19.81 |
| 2011 |  |  |  |  | 38367.30 |  | 21.04 | 13.41 | 22.88 | 4.58 | 18.31 |
| 2012 |  |  |  |  | 35132.20 |  | 24.20 | 13.29 | 23.95 | 2.27 | 21.67 |
| **Latvia** | | | | | | | | | | | |
| 2000 | 50.60 | 8.70 | 14.40 | 5.70 | 3308.50 | 2.60 | 20.37 | 7.54 | 16.11 | -2.36 | 18.47 |
| 2001 | 46.40 | 7.80 | 13.10 | 7.30 | 3556.90 | 2.50 | 25.37 | 6.59 | 20.82 | -6.76 | 27.58 |
| 2002 | 43.00 | 8.70 | 12.00 | 7.20 | 4032.10 | 1.90 | 18.17 | 7.01 | 19.53 | -8.10 | 27.63 |
| 2003 | 39.50 | 7.30 | 10.60 | 7.60 | 4889.30 | 2.90 | 18.88 | 6.32 | 20.02 | -5.69 | 25.71 |
| 2004 | 36.70 | 6.00 | 10.40 | 8.90 | 6080.80 | 6.20 | 20.66 | 6.31 | 17.64 | -6.95 | 24.59 |
| 2005 | 36.20 | 6.30 | 9.00 | 10.10 | 7165.40 | 6.70 | 7.20 | 6.25 | 18.38 | -6.18 | 24.57 |
| 2006 | 32.60 | 4.40 | 6.80 | 11.20 | 8986.40 | 6.50 | 6.48 | 6.95 | 19.70 | -7.47 | 27.17 |
| 2007 | 29.20 | 5.50 | 6.00 | 9.60 | 13073.40 | 10.10 | 9.63 | 7.14 | 18.09 | -8.61 | 26.70 |
| 2008 | 34.40 | 5.70 | 7.50 | -3.30 | 15463.70 | 15.40 | 9.18 | 7.72 | 17.45 | -1.01 | 18.46 |
| 2009 | 33.50 | 5.70 | 16.90 | -17.70 | 12082.10 | 3.50 | 22.67 | 6.59 | 17.67 | -4.07 | 21.74 |
| 2010 | 30.40 | 3.50 | 18.70 | -0.90 | 11446.50 | -1.10 | 23.98 | 5.78 | 21.74 | -11.84 | 33.58 |
| 2011 | 32.10 | 4.60 | 15.40 | 5.50 | 13827.40 | 4.40 | 17.91 | 7.31 | 20.23 | -9.06 | 29.29 |
| 2012 | 33.10 | 4.50 |  |  | 13947.00 |  | 24.92 | 6.26 | 18.61 | -9.25 | 27.86 |
| **Lithouania** | | | | | | | | | | | |
| 2000 | 75.10 | 14.00 | 16.40 | 3.62 | 3267.30 | 1.40 | 20.92 | 8.01 | 16.06 | -2.52 | 18.58 |
| 2001 | 71.20 | 12.10 | 17.40 | 6.70 | 3503.30 | 2.00 | 23.80 | 7.04 | 20.84 | -6.04 | 26.88 |
| 2002 | 74.90 | 10.10 | 13.80 | 6.84 | 4113.80 | -1.00 | 19.78 | 7.61 | 19.94 | -8.24 | 28.18 |
| 2003 | 68.30 | 11.20 | 12.40 | 10.28 | 5448.80 | -1.30 | 19.35 | 6.70 | 19.88 | -5.72 | 25.59 |
| 2004 | 64.40 | 11.40 | 11.40 | 7.37 | 6709.70 | 2.90 | 22.43 | 6.66 | 17.86 | -7.23 | 25.10 |
| 2005 | 62.20 | 10.00 | 8.30 | 7.79 | 7851.00 | 3.00 | 18.98 | 6.76 | 18.66 | -5.55 | 24.21 |
| 2006 | 48.60 | 8.40 | 5.60 | 7.81 | 9249.90 | 4.50 | 17.01 | 7.32 | 20.47 | -7.25 | 27.72 |
| 2007 | 50.00 | 7.60 | 4.30 | 9.80 | 12170.40 | 8.10 | 22.55 | 7.72 | 18.50 | -7.11 | 25.61 |
| 2008 | 54.40 | 8.50 | 5.80 | 2.91 | 14832.70 | 8.50 | 19.31 | 8.12 | 17.74 | -0.76 | 18.50 |
| 2009 | 55.70 | 8.30 | 13.70 | -14.85 | 11713.90 | 1.30 | 22.10 | 6.97 | 18.14 | -3.53 | 21.67 |
| 2010 | 49.50 | 8.40 | 17.80 | 1.60 | 11852.20 | 3.80 | 27.15 | 6.37 | 21.76 | -10.72 | 32.48 |
| 2011 |  |  | 15.40 | 6.05 | 14227.70 | 3.40 | 21.05 | 7.69 | 19.73 | -7.59 | 27.32 |
| 2012 |  |  |  |  | 14172.30 |  | 25.19 | 6.86 | 19.27 | -9.08 | 28.36 |
| **Montenegro** | | | | | | | | | | | |
| 2000 | 0.00 | 0.00 | 19.20 | 1.00 | 1610.40 | 24.80 | 27.62 | 9.18 | 19.58 | -4.78 | 24.37 |
| 2001 |  |  | 21.20 | 1.10 | 1896.70 | 28.00 | 25.38 | 8.71 | 18.85 | -4.49 | 23.34 |
| 2002 |  |  | 20.70 | 1.90 | 2097.70 | 9.40 | 25.89 | 8.95 | 18.44 | -2.95 | 21.38 |
| 2003 |  |  | 22.70 | 2.50 | 2783.70 | 6.70 | 21.26 | 8.28 | 19.58 | -4.78 | 24.37 |
| 2004 |  |  | 27.70 | 4.40 | 3373.00 | 4.30 | 27.64 | 8.15 | 17.84 | -2.50 | 20.33 |
| 2005 | 23.90 | 9.00 | 30.30 | 4.20 | 3665.30 | 1.80 | 26.99 | 7.53 | 17.98 | -3.90 | 21.88 |
| 2006 | 0.00 | 0.00 | 29.60 | 8.60 | 4370.60 | 2.00 | 24.69 | 8.15 | 17.37 | -3.62 | 20.99 |
| 2007 | 26.30 | 7.50 | 19.40 | 10.70 | 5945.90 | 8.00 | 25.80 | 9.13 | 20.08 | -1.85 | 21.93 |
| 2008 | 23.80 | 11.30 | 16.80 | 6.90 | 7335.90 | 7.30 | 22.02 | 9.22 | 18.95 | 0.25 | 18.71 |
| 2009 | 21.90 | 7.30 | 19.10 | 5.70 | 6713.10 | 1.50 | 29.07 | 8.90 | 18.48 | -1.16 | 19.64 |
| 2010 |  |  |  |  | 6636.10 |  | 28.94 | 9.24 | 19.34 | -0.18 | 19.53 |
| 2011 |  |  |  |  | 7253.40 |  | 19.33 | 9.02 | 19.31 | -0.33 | 19.65 |
| 2012 |  |  |  |  | 6514.10 |  | 24.26 | 9.49 | 21.14 | -4.33 | 25.47 |
| **Netherlands** | | | | | | | | | | | |
| 2000 | 11.10 | 5.10 | 2.60 | 7.00 | 25958.20 | 2.60 | 30.88 | 10.68 | 17.24 | 4.15 | 13.09 |
| 2001 | 10.90 | 4.80 | 2.00 | 2.10 | 26554.10 | 4.50 | 33.03 | 10.14 | 18.40 | 2.47 | 15.93 |
| 2002 | 11.50 | 5.10 | 2.30 | 0.10 | 28762.20 | 3.40 | 30.68 | 10.56 | 18.74 | 2.33 | 16.41 |
| 2003 | 10.90 | 4.70 | 5.40 | 0.90 | 35186.90 | 2.10 | 21.99 | 10.21 | 19.17 | 1.35 | 17.83 |
| 2004 | 10.90 | 4.80 | 6.40 | 3.50 | 39680.50 | 1.20 | 29.83 | 10.18 | 18.77 | 3.06 | 15.71 |
| 2005 | 11.40 | 5.00 | 6.50 | 3.30 | 41198.70 | 1.70 | 26.70 | 10.38 | 17.59 | 2.09 | 15.50 |
| 2006 | 11.10 | 4.80 | 5.50 | 5.50 | 44009.00 | 1.10 | 25.92 | 10.91 | 21.92 | 1.07 | 20.85 |
| 2007 | 9.90 | 4.10 | 4.50 | 4.60 | 50858.50 | 1.60 | 32.04 | 10.95 | 17.25 | 3.64 | 13.61 |
| 2008 | 10.30 | 4.40 | 3.80 | 2.00 | 56630.80 | 2.50 | 27.64 | 10.35 | 17.90 | 2.17 | 15.73 |
| 2009 | 10.90 | 4.50 | 4.80 | -5.00 | 51906.50 | 1.20 | 25.92 | 10.26 | 18.44 | 0.51 | 17.94 |
| 2010 | 11.40 | 4.60 | 5.40 | 4.90 | 50338.30 | 1.30 | 28.06 | 8.81 | 19.85 | -1.68 | 21.53 |
| 2011 | 11.50 | 5.10 | 5.40 | 2.10 | 53540.60 | 2.30 | 27.25 | 10.64 | 16.85 | 3.05 | 13.80 |
| 2012 |  |  |  |  | 49128.10 |  | 29.44 | 10.04 | 18.37 | 0.41 | 17.96 |
| **Norway** | | | | | | | | | | | |
| 2000 | 17.50 | 5.40 | 3.40 | 2.60 | 37472.70 | 3.00 | 38.01 | 2.31 | 11.38 | -5.25 | 16.62 |
| 2001 | 16.90 | 5.80 | 3.60 | 1.50 | 37867.10 | 2.70 | 33.94 | 1.14 | 11.59 | -9.81 | 21.40 |
| 2002 | 14.70 | 5.40 | 3.90 | 0.90 | 42291.80 | 0.80 | 29.74 | 1.97 | 13.89 | -8.28 | 22.17 |
| 2003 | 15.50 | 5.20 | 4.50 | 0.40 | 49263.50 | 2.00 | 32.97 | 2.21 | 14.29 | -8.57 | 22.86 |
| 2004 | 14.70 | 6.80 | 4.40 | 3.40 | 56627.70 | 0.60 | 34.90 | 2.20 | 12.04 | -7.67 | 19.71 |
| 2005 | 14.70 | 6.90 | 4.60 | 1.90 | 65767.00 | 1.50 | 35.74 | 2.26 | 13.09 | -5.66 | 18.76 |
| 2006 | 15.70 | 5.60 | 3.40 | 1.40 | 72959.70 | 2.50 | 34.59 | 2.65 | 13.12 | -8.22 | 21.35 |
| 2007 | 13.20 | 5.60 | 2.50 | 1.70 | 83556.30 | 0.70 | 37.14 | 2.15 | 11.76 | -9.10 | 20.86 |
| 2008 | 13.30 | 6.10 | 2.50 | -1.30 | 95189.90 | 3.40 | 34.53 | 2.10 | 12.57 | -5.19 | 17.76 |
| 2009 | 15.30 | 6.10 | 3.10 | -2.80 | 78457.40 | 2.30 | 32.54 | 1.83 | 11.95 | -8.19 | 20.15 |
| 2010 | 14.20 | 5.90 | 3.50 | -0.80 | 86096.10 | 2.30 | 27.77 | 0.10 | 12.31 | -10.78 | 23.09 |
| 2011 | 15.80 | 6.20 | 3.30 | -0.10 | 99091.10 | 1.20 | 41.97 | 2.71 | 12.47 | -9.14 | 21.61 |
| 2012 | 13.10 | 5.20 |  |  | 99635.90 |  | 34.89 | 1.10 | 10.74 | -9.48 | 20.22 |
| **Poland** | | | | | | | | | | | |
| 2000 | 24.10 | 4.20 | 15.10 | 4.00 | 4488.10 | 8.30 | 19.25 | 9.40 | 17.66 | -1.32 | 18.98 |
| 2001 | 24.40 | 3.70 | 17.50 | 1.30 | 4991.10 | 3.60 | 22.28 | 8.14 | 19.69 | -3.57 | 23.26 |
| 2002 | 23.80 | 4.10 | 18.00 | 1.30 | 5196.90 | 0.80 | 19.74 | 9.02 | 20.12 | -5.37 | 25.49 |
| 2003 | 23.70 | 3.70 | 20.00 | 3.60 | 5693.40 | 1.60 | 15.14 | 8.14 | 19.23 | -4.51 | 23.74 |
| 2004 | 24.50 | 3.80 | 19.00 | 5.20 | 6639.90 | 4.40 | 19.03 | 8.14 | 18.55 | -4.79 | 23.34 |
| 2005 | 24.20 | 3.80 | 17.60 | 3.30 | 7976.10 | 0.80 | 17.76 | 8.26 | 19.39 | -2.86 | 22.26 |
| 2006 | 23.10 | 3.50 | 14.80 | 6.00 | 9001.80 | 1.40 | 17.48 | 8.73 | 21.78 | -6.98 | 28.76 |
| 2007 | 20.90 | 3.30 | 11.20 | 6.70 | 11252.40 | 4.20 | 21.73 | 9.38 | 18.58 | -0.08 | 18.66 |
| 2008 | 22.60 | 3.30 | 9.50 | 5.10 | 13906.20 | 3.30 | 18.88 | 9.42 | 18.75 | 1.12 | 17.63 |
| 2009 | 25.50 | 3.80 | 12.10 | 1.80 | 11440.60 | 3.80 | 21.13 | 8.58 | 19.28 | -2.79 | 22.07 |
| 2010 | 24.80 | 3.30 | 12.40 | 3.70 | 12484.10 | 2.90 | 25.29 | 7.56 | 21.05 | -7.40 | 28.45 |
| 2011 | 24.00 | 3.00 |  |  | 13607.70 |  | 17.29 | 8.98 | 18.60 | -3.55 | 22.15 |
| 2012 |  |  |  |  | 12876.50 |  | 19.14 | 8.56 | 19.80 | -5.97 | 25.77 |
| **Portugal** | | | | | | | | | | | |
| 2000 | 6.40 | 1.40 | 3.90 | 3.92 | 11502.40 | 2.85 | 34.03 | 15.41 | 22.59 | 6.55 | 16.04 |
| 2001 | 9.30 | 2.30 | 4.00 | 1.97 | 11729.10 | 4.37 | 35.26 | 15.64 | 22.70 | 7.47 | 15.23 |
| 2002 | 14.70 | 3.70 | 5.00 | 0.76 | 12882.30 | 3.60 | 29.91 | 15.78 | 22.45 | 10.09 | 12.36 |
| 2003 | 13.50 | 3.50 | 6.30 | -0.91 | 15772.70 | 3.22 | 27.78 | 16.05 | 25.12 | 8.91 | 16.20 |
| 2004 |  |  | 6.60 | 1.56 | 18046.40 | 2.37 | 16.82 | 15.69 | 23.50 | 8.72 | 14.78 |
| 2005 |  |  | 7.60 | 0.78 | 18784.50 | 2.28 | 14.61 | 15.63 | 24.12 | 7.55 | 16.56 |
| 2006 |  |  | 7.70 | 1.45 | 19820.40 | 3.11 | 28.76 | 16.23 | 24.61 | 7.43 | 17.18 |
| 2007 | 10.60 | 3.40 | 8.00 | 2.37 | 22778.90 | 2.45 | 16.93 | 15.41 | 22.34 | 8.62 | 13.71 |
| 2008 | 11.30 | 2.90 | 7.60 | -0.01 | 24816.50 | 2.59 | 22.74 | 15.44 | 22.44 | 8.72 | 13.72 |
| 2009 | 11.60 | 2.70 | 9.40 | -2.91 | 23062.60 | -0.83 | 26.01 | 16.33 | 24.27 | 8.43 | 15.85 |
| 2010 | 11.50 | 3.30 | 10.80 | 1.94 | 22538.70 | 1.40 | 37.36 | 16.00 | 25.32 | 9.25 | 16.07 |
| 2011 | 11.00 | 2.80 | 12.70 | 1.55 | 23196.20 | 3.70 | 23.96 | 16.47 | 22.62 | 9.50 | 13.12 |
| 2012 |  |  |  |  | 20732.60 |  | 17.91 | 15.71 | 23.17 | 8.43 | 14.74 |
| **Romania** | | | | | | | | | | | |
| 2000 | 19.10 | 3.60 | 7.20 | 2.50 | 1662.20 | 45.70 | 11.57 | 10.12 | 21.28 | -5.26 | 26.54 |
| 2001 | 18.70 | 3.10 | 7.20 | 5.80 | 1833.80 | 34.50 | 20.20 | 9.50 | 21.37 | -5.86 | 27.23 |
| 2002 | 21.10 | 3.90 | 7.50 | 8.00 | 2116.30 | 22.50 | 17.69 | 9.97 | 22.00 | -4.20 | 26.19 |
| 2003 | 19.60 | 3.70 | 6.80 | 5.50 | 2756.30 | 15.30 | 14.61 | 9.00 | 21.52 | -5.90 | 27.42 |
| 2004 | 18.60 | 3.10 | 8.00 | 8.80 | 3533.30 | 11.90 | 19.50 | 9.23 | 20.13 | -4.51 | 24.64 |
| 2005 | 17.40 | 3.20 | 7.20 | 4.40 | 4651.70 | 9.00 | 25.54 | 8.77 | 19.98 | -3.87 | 23.85 |
| 2006 | 18.10 | 3.30 | 7.30 | 8.10 | 5789.20 | 6.56 | 18.46 | 9.04 | 20.66 | -5.66 | 26.32 |
| 2007 | 15.90 | 3.10 | 6.40 | 6.50 | 8170.10 | 4.84 | 19.95 | 10.24 | 22.42 | -2.01 | 24.43 |
| 2008 | 16.20 | 3.10 | 5.80 | 7.50 | 9949.40 | 7.85 | 17.22 | 9.84 | 20.92 | -2.54 | 23.46 |
| 2009 | 17.60 | 2.90 | 6.90 | -6.40 | 8069.00 | 5.59 | 18.20 | 9.89 | 20.93 | -2.07 | 23.00 |
| 2010 | 18.30 | 3.30 | 7.30 | -1.50 | 8139.10 | 6.09 | 24.88 | 10.08 | 22.37 | -3.46 | 25.82 |
| 2011 | 17.20 | 2.70 | 7.04 | 2.50 | 9063.70 | 3.33 | 14.17 | 9.73 | 20.94 | -2.41 | 23.35 |
| 2012 |  |  |  |  | 8445.30 |  | 17.96 | 10.53 | 24.17 | -6.66 | 30.82 |
| **Serbia** | | | | | | | | | | | |
| 2000 | 23.50 | 8.40 | 12.10 | 5.30 | 870.10 | 1.13 | 13.82 | 11.40 | 22.58 | -3.46 | 26.05 |
| 2001 | 22.50 | 7.40 | 12.20 | 5.30 | 1634.90 | 0.39 | 25.47 | 10.41 | 21.48 | -4.23 | 25.71 |
| 2002 | 22.50 | 6.80 | 13.30 | 4.30 | 2148.90 | 0.15 | 22.13 | 10.99 | 21.61 | -1.52 | 23.13 |
| 2003 | 21.70 | 6.70 | 14.60 | 2.50 | 2832.50 | 0.11 | 18.38 | 10.21 | 22.69 | -4.30 | 26.99 |
| 2004 | 20.50 | 6.40 | 18.50 | 9.30 | 3331.20 | 0.14 | 25.52 | 10.04 | 20.40 | -2.24 | 22.63 |
| 2005 | 20.90 | 7.30 | 20.80 | 5.40 | 3528.10 | 0.18 | 26.12 | 9.37 | 20.25 | -3.36 | 23.61 |
| 2006 | 21.20 | 7.10 | 20.90 | 3.60 | 4129.80 | 0.07 | 22.51 | 10.11 | 21.08 | -2.89 | 23.97 |
| 2007 | 20.10 | 6.50 | 18.10 | 5.40 | 5458.10 | 0.10 | 23.95 | 11.30 | 23.04 | -0.82 | 23.86 |
| 2008 | 19.10 | 6.70 | 13.60 | 3.80 | 6701.80 | 0.09 | 19.70 | 11.23 | 21.29 | 0.48 | 20.82 |
| 2009 | 20.60 | 6.50 | 16.10 | -3.50 | 5821.30 | 0.07 | 25.19 | 10.98 | 21.16 | -1.11 | 22.27 |
| 2010 | 18.10 | 4.80 | 19.20 | 1.80 | 5399.30 | 0.12 | 27.76 | 11.08 | 21.57 | -0.35 | 21.93 |
| 2011 | 18.40 | 5.80 | 26.10 | 1.60 | 6422.70 | 0.07 | 15.14 | 10.79 | 21.71 | -0.02 | 21.73 |
| 2012 | 19.00 | 5.20 | 0.23 | -1.01 | 5666.20 | 1.20 | 19.53 | 11.40 | 23.94 | -4.91 | 28.85 |
| **Slovakia** | | | | | | | | | | | |
| 2000 | 20.60 | 4.10 | 18.80 |  | 5402.00 | 12.20 | 22.58 | 9.04 | 18.93 | -4.36 | 23.29 |
| 2001 | 20.20 | 3.40 | 19.30 | 3.50 | 5707.50 | 7.30 | 24.95 | 7.85 | 18.94 | -6.16 | 25.10 |
| 2002 | 21.10 | 3.00 | 18.70 | 4.60 | 6536.20 | 3.10 | 24.30 | 8.65 | 19.83 | -3.72 | 23.55 |
| 2003 | 21.90 | 3.00 | 17.60 | 4.80 | 8711.70 | 8.60 | 16.38 | 8.07 | 19.71 | -4.95 | 24.65 |
| 2004 | 18.80 | 3.30 | 18.20 | 5.00 | 10671.30 | 7.60 | 23.63 | 7.69 | 17.66 | -5.09 | 22.75 |
| 2005 | 19.30 | 2.80 | 16.30 | 6.70 | 11665.50 | 2.70 | 25.80 | 7.43 | 18.46 | -4.59 | 23.05 |
| 2006 | 0.00 | 0.00 | 13.40 | 8.50 | 13111.80 | 4.50 | 20.79 | 8.03 | 20.62 | -6.43 | 27.05 |
| 2007 | 0.00 | 0.00 | 11.10 | 10.60 | 16006.90 | 2.80 | 24.55 | 9.05 | 19.59 | -2.37 | 21.96 |
| 2008 | 16.40 | 2.40 | 9.50 | 5.80 | 18558.90 | 4.60 | 22.33 | 9.05 | 18.25 | 0.03 | 18.22 |
| 2009 | 17.20 | 1.60 | 12.00 | -4.90 | 16455.20 | 1.60 | 24.04 | 8.64 | 19.47 | -3.71 | 23.18 |
| 2010 | 17.20 | 2.70 | 14.40 | 4.20 | 16509.90 | 1.00 | 34.98 | 8.18 | 20.26 | -3.90 | 24.15 |
| 2011 |  |  |  |  | 18065.70 |  | 17.44 | 8.91 | 19.46 | -2.12 | 21.58 |
| 2012 |  |  |  |  | 17151.20 |  | 19.87 | 9.01 | 20.49 | -5.84 | 26.33 |
| **Slovenia** | | | | | | | | | | | |
| 2000 | 38.10 | 11.60 | 11.80 | -0.20 | 10227.30 | 10.60 | 45.22 | 10.40 | 20.03 | -2.66 | 22.68 |
| 2001 | 39.60 | 8.80 | 11.20 | -0.50 | 10479.60 | 9.70 | 42.12 | 9.52 | 20.23 | -3.11 | 23.33 |
| 2002 | 36.00 | 7.80 | 11.30 | -0.60 | 11813.80 | 7.40 | 44.22 | 10.12 | 19.33 | -0.69 | 20.02 |
| 2003 | 36.90 | 8.40 | 10.90 | -1.20 | 14881.00 | 6.50 | 34.02 | 9.70 | 22.26 | -3.10 | 25.36 |
| 2004 | 30.20 | 10.40 | 10.30 | -0.30 | 17261.10 | 4.30 | 49.66 | 8.85 | 18.53 | -1.96 | 20.49 |
| 2005 | 32.20 | 7.70 | 10.20 | -0.40 | 18168.10 | 2.60 | 45.37 | 8.55 | 18.79 | -2.75 | 21.54 |
| 2006 | 33.00 | 7.90 | 9.40 | 0.40 | 19725.00 | 2.60 | 37.61 | 9.46 | 21.30 | -3.27 | 24.57 |
| 2007 | 25.90 | 6.80 | 7.70 | 0.60 | 23841.30 | 3.60 | 40.72 | 10.16 | 19.80 | -0.83 | 20.63 |
| 2008 | 25.30 | 5.70 | 6.70 | 1.70 | 27501.80 | 6.90 | 49.07 | 9.93 | 19.22 | 0.89 | 18.33 |
| 2009 | 26.70 | 6.10 | 9.10 | 1.50 | 24633.80 | -0.60 | 48.87 | 9.87 | 19.89 | -2.28 | 22.16 |
| 2010 | 25.80 | 5.00 | 10.50 | 1.80 | 23417.60 | 1.10 | 55.61 | 8.89 | 20.64 | -2.75 | 23.39 |
| 2011 |  |  | 11.50 | 1.60 | 24964.80 | 0.20 | 33.82 | 9.89 | 20.26 | 0.15 | 20.11 |
| 2012 |  |  |  |  | 22488.40 |  | 44.51 | 10.04 | 20.68 | -3.24 | 23.93 |
| **Spain** | | | | | | | | | | | |
| 2000 | 10.40 | 2.90 | 14.00 | 5.00 | 14787.80 | 3.96 | 20.24 | 13.66 | 22.49 | 3.85 | 18.64 |
| 2001 | 9.60 | 2.70 | 10.63 | 3.70 | 15359.10 | 2.71 | 18.51 | 13.90 | 23.35 | 4.27 | 19.09 |
| 2002 | 9.90 | 2.90 | 11.30 | 2.70 | 17019.50 | 4.00 | 20.44 | 13.93 | 22.05 | 7.03 | 15.02 |
| 2003 | 10.00 | 2.90 | 11.30 | 3.10 | 21495.70 | 2.60 | 20.29 | 14.27 | 24.93 | 5.49 | 19.44 |
| 2004 | 9.90 | 2.90 | 10.40 | 3.20 | 24919.70 | 3.20 | 16.72 | 13.66 | 22.78 | 6.14 | 16.64 |
| 2005 | 9.30 | 2.70 | 9.16 | 3.60 | 26510.10 | 3.70 | 13.19 | 13.60 | 23.57 | 4.01 | 19.55 |
| 2006 | 8.90 | 2.40 | 8.51 | 4.10 | 28481.20 | 2.70 | 18.89 | 14.43 | 24.81 | 4.61 | 20.19 |
| 2007 | 8.40 | 2.60 | 8.65 | 3.50 | 32707.70 | 4.20 | 16.08 | 13.52 | 22.35 | 5.92 | 16.43 |
| 2008 | 9.10 | 2.50 | 11.55 | 0.90 | 35580.10 | 1.40 | 20.44 | 13.47 | 22.76 | 5.55 | 17.21 |
| 2009 | 9.00 | 2.40 | 18.10 | -3.70 | 32331.50 | 0.80 | 18.14 | 14.32 | 24.03 | 5.03 | 18.99 |
| 2010 | 8.10 | 2.20 | 20.10 | -0.30 | 30736.00 | 3.00 | 24.12 | 13.48 | 24.33 | 5.40 | 18.93 |
| 2011 | 8.00 | 2.30 |  |  | 31975.00 |  | 16.06 | 14.70 | 23.35 | 6.21 | 17.14 |
| 2012 |  |  |  |  | 28985.30 |  | 15.54 | 13.91 | 24.02 | 4.48 | 19.54 |
| **Sweden** | | | | | | | | | | | |
| 2000 | 15.10 | 6.10 | 4.70 | 4.50 | 29283.00 | 1.00 | 27.51 | 3.66 | 13.60 | -5.29 | 18.90 |
| 2001 | 15.60 | 6.30 | 5.80 | 1.30 | 26969.20 | 2.40 | 23.93 | 2.33 | 14.58 | -10.12 | 24.71 |
| 2002 | 16.30 | 5.80 | 6.00 | 2.50 | 29571.70 | 2.20 | 19.30 | 3.07 | 16.36 | -9.57 | 25.93 |
| 2003 | 14.50 | 6.40 | 6.60 | 2.30 | 36961.40 | 1.90 | 19.50 | 3.07 | 17.08 | -10.43 | 27.51 |
| 2004 | 15.60 | 5.90 | 7.40 | 4.20 | 42442.30 | 0.40 | 21.78 | 2.84 | 13.75 | -9.26 | 23.01 |
| 2005 | 15.30 | 7.00 | 7.80 | 3.20 | 43085.40 | 0.50 | 21.69 | 3.27 | 15.69 | -6.84 | 22.52 |
| 2006 | 14.70 | 7.20 | 7.10 | 4.30 | 46256.20 | 1.40 | 23.03 | 3.62 | 15.86 | -8.38 | 24.24 |
| 2007 | 14.70 | 6.00 | 6.10 | 3.30 | 53324.60 | 2.20 | 21.66 | 3.29 | 13.92 | -9.42 | 23.34 |
| 2008 | 15.40 | 5.90 | 6.20 | -0.60 | 55746.80 | 3.40 | 22.99 | 3.40 | 14.84 | -4.62 | 19.46 |
| 2009 | 16.10 | 6.40 | 8.30 | -5.00 | 46206.90 | -0.30 | 21.73 | 2.70 | 14.15 | -10.00 | 24.16 |
| 2010 | 14.80 | 5.40 | 8.60 | 6.60 | 52076.30 | 1.30 | 21.16 | 0.89 | 15.61 | -12.58 | 28.20 |
| 2011 |  |  | 7.80 | 3.70 | 59593.30 | 2.60 | 24.70 | 3.84 | 15.67 | -10.73 | 26.40 |
| 2012 |  |  |  |  | 57134.10 |  | 23.30 | 2.18 | 13.78 | -10.25 | 24.03 |
| **Switzerland** | | | | | | | | | | | |
| 2000 | 23.30 | 8.20 | 1.80 | 3.90 | 37813.20 | 1.60 | 46.25 | 6.25 | 15.02 | -3.26 | 18.28 |
| 2001 | 22.00 | 7.90 | 1.70 | 1.40 | 38538.60 | 1.00 | 49.61 | 5.58 | 15.19 | -3.82 | 19.01 |
| 2002 | 22.40 | 9.10 | 2.50 | 0.10 | 41336.70 | 0.60 | 53.00 | 6.21 | 14.64 | -1.97 | 16.60 |
| 2003 | 19.10 | 7.80 | 3.70 | 0.00 | 47960.60 | 0.60 | 32.48 | 6.28 | 18.06 | -5.21 | 23.27 |
| 2004 | 18.80 | 7.90 | 3.90 | 2.80 | 53256.00 | 0.80 | 39.33 | 5.49 | 14.26 | -3.02 | 17.28 |
| 2005 | 19.40 | 7.60 | 3.80 | 3.00 | 54798.60 | 1.20 | 36.75 | 5.15 | 14.16 | -5.77 | 19.93 |
| 2006 | 18.40 | 8.20 | 3.30 | 4.00 | 57346.90 | 1.10 | 40.21 | 6.06 | 17.83 | -4.57 | 22.40 |
| 2007 | 19.10 | 8.00 | 2.80 | 4.10 | 63225.30 | 0.70 | 45.55 | 6.22 | 13.63 | -2.48 | 16.11 |
| 2008 | 17.50 | 8.00 | 2.60 | 2.30 | 72120.20 | 2.40 | 44.89 | 5.74 | 13.98 | -2.59 | 16.57 |
| 2009 | 17.10 | 5.50 | 3.70 | -2.10 | 69669.30 | -0.50 | 37.47 | 5.92 | 15.94 | -4.68 | 20.63 |
| 2010 | 14.50 | 5.50 | 3.50 | 3.00 | 74276.70 | 0.70 | 40.75 | 5.03 | 16.26 | -5.00 | 21.26 |
| 2011 |  |  | 2.80 | 1.80 | 87998.40 | 0.20 | 37.73 | 7.09 | 15.72 | -2.27 | 18.00 |
| 2012 |  |  | 2.90 | 1.10 | 83295.30 | -0.70 | 50.53 | 6.19 | 15.98 | -6.44 | 22.41 |
| **UK** | | | | | | | | | | | |
| 2000 | 10.30 | 2.90 | 5.70 | 4.46 | 26296.40 | 1.10 | 41.05 | 9.15 | 15.27 | 4.41 | 10.86 |
| 2001 | 10.20 | 2.60 | 5.10 | 3.15 | 25864.40 | 0.80 | 30.93 | 8.81 | 15.35 | 2.62 | 12.72 |
| 2002 | 10.10 | 2.80 | 5.00 | 2.66 | 28202.90 | 1.50 | 38.93 | 9.46 | 15.71 | 4.84 | 10.87 |
| 2003 | 9.60 | 2.70 | 5.10 | 3.52 | 32586.60 | 1.30 | 26.11 | 9.50 | 16.52 | 3.48 | 13.04 |
| 2004 | 9.80 | 2.90 | 4.70 | 2.96 | 38309.80 | 1.50 | 35.03 | 9.46 | 16.10 | 4.38 | 11.72 |
| 2005 | 9.30 | 2.80 | 4.70 | 2.09 | 39934.90 | 2.10 | 31.06 | 9.43 | 15.40 | 3.74 | 11.66 |
| 2006 | 9.60 | 2.60 | 5.10 | 2.61 | 42447.80 | 2.70 | 33.82 | 9.76 | 18.01 | 3.54 | 14.47 |
| 2007 | 9.10 | 2.40 | 5.50 | 3.47 | 48322.70 | 2.10 | 34.90 | 9.64 | 14.37 | 4.49 | 9.88 |
| 2008 | 9.90 | 2.70 | 5.10 | -1.10 | 45170.50 | 4.10 | 37.99 | 9.09 | 15.37 | 3.30 | 12.08 |
| 2009 | 9.80 | 2.60 | 6.70 | -4.37 | 37075.50 | 1.90 | 34.66 | 9.25 | 15.47 | 2.27 | 13.20 |
| 2010 | 9.40 | 2.70 | 7.90 | 2.09 | 38363.40 | 3.30 | 26.73 | 8.02 | 15.71 | -0.80 | 16.50 |
| 2011 |  |  |  |  | 40972.00 |  | 33.13 | 9.71 | 14.23 | 3.23 | 10.99 |
| 2012 |  |  |  |  | 41050.80 |  | 33.64 | 8.97 | 15.51 | 4.25 | 11.25 |

**Table A:** The complete dataset of the current study

1. **Statistical analysis**

Three separate analyses were performed with the use of suicide data in combination with climate or economic variables only while the third included both economic and climate variables and genders were considered separately also. Figure A shows a flow diagram of our analysis steps.

Initially, hierarchical cluster analysis [1] was applied separately for the economy and climate variables and indicated groups (clusters) of strongly related variables. Next, a first layer of Principal Components Analysis [2] was used to select the most prominent variable within each identified cluster (from cluster analysis). Then, an additional second layer of PCA utilized only these prominent variables and uncovered the underlying factor structure and attributed factor scores to each case. This sequence of one cluster analysis and two PCAs yielded the respective independent variables (factor scores) which were used in the subsequent regression analysis.

This method for the exploration of the underlying structure and the identification of the respective ‘hidden’ variables has been previously described and utilized [3].

The term ‘emerging factor’ which will be used below corresponds to the weighted sums of the PCA factor scores, with regression coefficients as weights after regression analysis was performed.

As already mentioned, in this paper we approach the investigation of relationship between suicidality, economy and climate through regression modeling. Suicidality rates are used as dependent variables while the previously obtained economy and climate dimensions (after cluster analysis and PCAs) are employed as predictors. In order to facilitate robustness to nonlinearities, within the framework of regression analysis, we relaxed the constraint on strict linear relations between variables by anticipating necessary optimal transformations of the original variables. As optimal scaling algorithms are now readily available, we employed CATREG (Categorical Regression) [4] as the main tool for optimally scaling and transforming the regression variables alongside with computation of regression coefficients, followed by a typical linear multiple regression analysis of composite economy and climate factors which are formed by the respective and optimally scaled/transformed variables.

| **Cluster Analysis**  *(separately for the* ***economy*** *and* ***climate*** *categories of variables)*  **PCA (1^st^ layer)**  *(within each cluster)*  **PCA (2^nd^ layer)**  *(with prominent variables, separately for the* ***economy*** *and* ***climate*** *categories of variables)*  **CATREG**  *(regression between suicidality (DV) and orthogonal dimensions (IVs), with concurrent optimal transformation of IVs)*  **Linear Multiple Regression**  *(suicidality as DV and emerging* ***economy*** *and* ***climate*** *factors as IVs)*  Clusters of variables    Prominent variable of each cluster    Orthogonal dimensions of the **economy** and **climate** categories of variables    Regression coefficients for optimally transformed IV and formation of emerging **economy** and **climate** factors |
| --- |

**Figure A:** Flow diagram of our analysis with intermediate outcomes.

Table B shows the available Suicidality, economy and climate variables which were included in the analysis of the current paper. In addition, to examine the possible retrospective character of the economy and climate in their relation to suicidality, we also constructed time-lagged and time-averaged versions for each one of the above variables and included these in our analysis together with the original variables. More specifically, lag1 and lag2 variables were created by shifting forward each year’s values by 1 and 2 years respectively. For example, a lag2 variable’s value for year 2005 is the year’s 2003 original variable’s value. In a similar way, 3-years’ time-averaged variables (designated as _ma3) represent the value obtained from the mean of values of the current and two past years. For example, the value for ma3 in year 2005 is replaced by the mean of the original variable’s values for years 2003, 2004 and 2005. Unavoidably, and since these constructed variables were computed from the available data of the 2000-2012 period only, it was not possible to obtain their complete profile over time. Nevertheless, our analysis still remains capable to cover a decade’s period.

| **Category** | **Variable name** | **Label** | **Details/Remarks** |
| --- | --- | --- | --- |
| Suicidality | Male suicides | *MSu* | Number of suicides per 100.000 of population |
|  | Female suicides | *FSu* | Number of suicides per 100.000 of population |
| Economy | National Unemployment rate | *NU* | in % |
|  | National Growth rate | *NG* | in % |
|  | GDP per capita | *GDP* | in USD |
|  | Inflation rate | *Infl.* | in % |
|  | National Unemployment rate_lag1 | *NU_lag1* | *National Unemployment rate* sequence, shifted forward 1 year (e.g. current year’s value=previous year’s original value) |
|  | National Growth rate_lag1 | *NG_lag1* | *National Growth rate* sequence, shifted forward 1 year (e.g. current year’s value=previous year’s original value) |
|  | GDP per capita_lag1 | *GDP_lag1* | *GDP per capita* sequence, shifted forward 1 year (e.g. current year’s value=previous year’s original value) |
|  | Inflation rate_lag1 | *Infl_lag1* | *Inflation rate* sequence, shifted forward 1 years (e.g. current year’s value=2^nd^ previous year’s original value) |
|  | National Unemployment rate_lag2 | *NU_lag2* | *National Unemployment rate* sequence, shifted forward 2 years (e.g. current year’s value=2^nd^ previous year’s original value) |
|  | National Growth rate_lag2 | *NG_lag2* | *National Growth rate* sequence, shifted forward 2 years (e.g. current year’s value=2^nd^ previous year’s original value) |
|  | GDP per capita_lag2 | *GDP_lag2* | *GDP per capita* sequence, shifted forward 2 years (e.g. current year’s value=2^nd^ previous year’s original value) |
|  | Inflation rate_lag2 | *Infl_lag2* | *Inflation rate* sequence, shifted forward 2 years (e.g. current year’s value=2^nd^ previous year’s original value) |
|  | National Unemployment rate_ma3 | *NU_ma3* | *National Unemployment rate* sequence, 3-years average (current, previous, 2^nd^ previous) |
|  | National Growth rate_ma3 | *NG_ma3* | *National Growth rate* sequence, 3-years average (current, previous, 2^nd^ previous) |
|  | GDP per capita_ma3 | *GDP_ma3* | *GDP per capita* sequence, 3-years average (current, previous, 2^nd^ previous) |
|  | Inflation rate_ma3 | *Infl_ma3* | *Inflation rate* time sequence, 3-years average (current, previous, 2^nd^ previous) |
| Climate | Rainfall | *Rfl.* | Annual average (in mm) |
|  | Temperature | *Temp.* | Annual average (in °C) |
|  | Monthly Max. Temperature | *MMT* | Maximum of monthly averaged temperatures (in °C) |
|  | Monthly Min. Temperature | *MmT* | Minimum of monthly averaged temperatures (in °C) |
|  | Annual Temperature Range | *ATR* | Difference between the maximum and minimum of monthly averaged temperatures (in °C) |
|  | Rainfall_lag1 | *Rfl_lag1* | *Rainfall* sequence, shifted forward 1 year (e.g. current year’s value=previous year’s original value) |
|  | Temperature_lag1 | *Temp_lag1* | *Temperature* sequence, shifted forward 1 year (e.g. current year’s value=previous year’s original value) |
|  | Monthly Max. Temperature_lag1 | *MMT_lag1* | *Monthly Max. Temperature* sequence, shifted forward 1 year (e.g. current year’s value=previous year’s original value) |
|  | Monthly Min. Temperature_lag1 | *MmT_lag1* | *Monthly Min. Temperature* sequence, shifted forward 1 years (e.g. current year’s value=previous year’s original value) |
|  | Annual Temperature Range_lag1 | *ATR_lag1* | *Annual Temperature Range* sequence, shifted forward 1 years (e.g. current year’s value=previous year’s original value) |
|  | Rainfall_lag2 | *Rfl_lag2* | *Rainfall* sequence, shifted forward 2 years (e.g. current year’s value=2^nd^ previous year’s original value) |
|  | Temperature_lag2 | *Temp_lag2* | *Temperature* sequence, shifted forward 2 years (e.g. current year’s value=2^nd^ previous year’s original value) |
|  | Monthly Max. Temperature_lag2 | *MMT_lag2* | *Monthly Max. Temperature* sequence, shifted forward 2 years (e.g. current year’s value=2^nd^ previous year’s original value) |
|  | Monthly Min. Temperature_lag2 | *MmT_lag2* | *Monthly Min. Temperature* sequence, shifted forward 2 years (e.g. current year’s value=2^nd^ previous year’s original value) |
|  | Annual Temperature Range_lag2 | *ATR_lag2* | *Annual Temperature Range* sequence, shifted forward 1 years (e.g. current year’s value=2^nd^ previous year’s original value) |
|  | Rainfall_ma3 | *Rfl_ma3* | *Rainfall* sequence, 3-years average (current, previous, 2^nd^ previous) |
|  | Temperature_ ma3 | *Temp_ ma3* | *Temperature* sequence, 3-years average (current, previous, 2^nd^ previous) |
|  | Monthly Max. Temperature_ ma3 | *MMT_ ma3* | *Monthly Max. Temperature* sequence, 3-years average (current, previous, 2^nd^ previous) |
|  | Monthly Min. Temperature_ ma3 | *MmT_ma3* | *Monthly Min. Temperature* sequence, 3-years average (current, previous, 2^nd^ previous) |
|  | Annual Temperature Range_ ma3 | *ATR_ ma3* | *Annual Temperature Range* sequence, 3-years average (current, previous, 2^nd^ previous) |

**Table B:** Data variables which were used in our analysis, together with their brief notation and descriptions.

1. **Results**

*5.a Cluster Analysis*

Cluster analysis was used to reveal the structure of homologous variables (economy or climate standardized variables). Hierarchical clustering with centroid linkage based on squared Euclidean distances identified the major clusters. By visually inspecting the obtained dendrograms (Figures B and C) we could identify 4 clusters (one for each of the macroeconomic quantities GDP, NU, etc.) for the economy category of variables, and 5 clusters for the climate category, one for each climate quantity, as previously. As expected, similar quantities had formed more cohesive clusters. Such a finer clustering, which actually reflects the major types of quantities (e.g. *GDP*, *Infl.*, *NU*, etc.), was chosen for the initial steps of our analysis so as to evidence and facilitate proper subgrouping of variables at the most atomic and homogeneous level of the analysis concept.


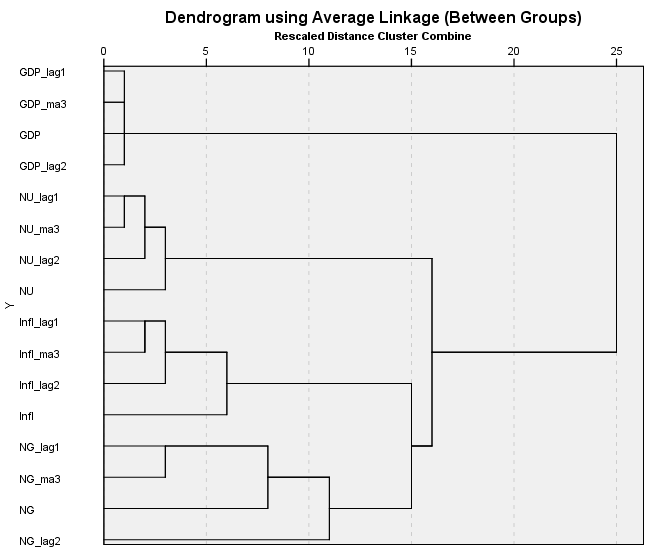


**Figure B:** Dendrogram of cluster analyses for the economy category of variables. Similar quantities form simple clusters (e.g. GDP, NU, etc.), which group together in higher levels of agglomeration. We may identify 4 simple clusters.


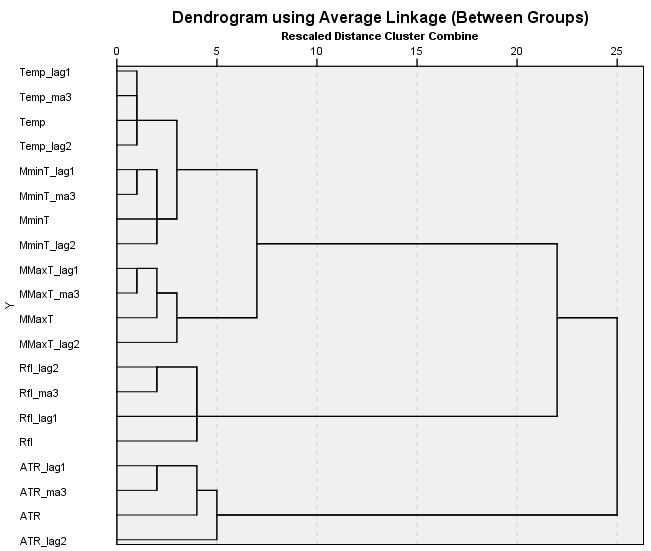


**Figure C:** Dendrogram of cluster analyses for the climate category of variables. Similar quantities form simple clusters (e.g. Temp, MmT, etc.), which group together in higher levels of agglomeration. We may identify 5 simple clusters.

*5b PCA*

We chose to perform the first layer of PCA at this finer level of clustering. Within each cluster, a 1-factor solution was identified, since (as expected) the correlation between the cluster’s variables was very high. We chose as the cluster’s prominent variable the one which had the highest loading (maximum loading equal to 1, while the remaining variables’ loadings were ~0.995). This variable had also the highest Pearson correlations with the rest of the other variables.

As it turned out, for all clusters, the variables (see table B) describing the 3-year average were identified as the most representative ones, e.g. *GDP_ma3*, *NU_ma3*, etc.

Next, all the previously chosen prominent variables were subjected to a second layer of PCA, separately for the economy and climate categories of variables. For both, PCA scree plots showed (based on a compromise between Kaiser’s eigenvalue-one criterion and Catell’s Scree test [5], we adopted an eigenvalue threshold >0.75 to retain considerable explained variance’s components) that 2-3 orthogonal dimensions might be extracted with 93% explained variance for the economy category (3 dimensions), and 91.3% for the climate category (2 dimensions) (Figures D and E) Tables C and D show the obtained dimensions for the economy and climate categories after Varimax orthogonal rotation, where component loadings >.7 are selected as strong. For the economy category, *NU_ma3* and *GDP_ma3* constitute the highest explained variance dimension (1^st^ dimension), and *Infl_ma3* and *NG_ma3* the 2^nd^ and 3^rd^ dimensions respectively. For the climate category, *Temp_ma3*, *MMT_ma3* and *MmT_ma3* constitute the 1^st^ dimension, while *Rfl_ma3* and *ATR_ma3* load mostly on the 2^nd^ dimension.


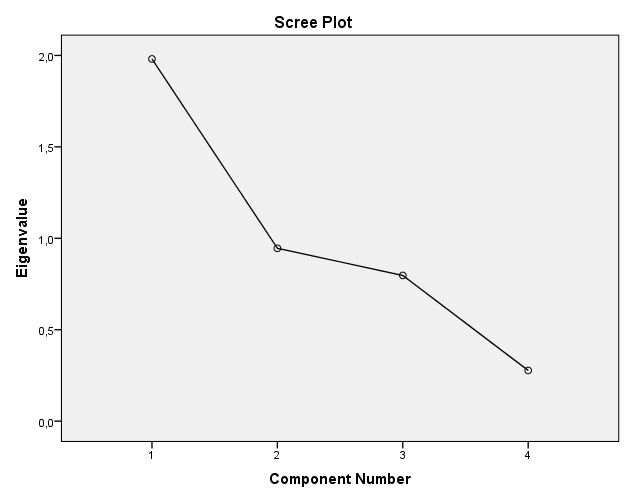


**Figure D:** eigenvalue plots (scree plots) for the economy PCA


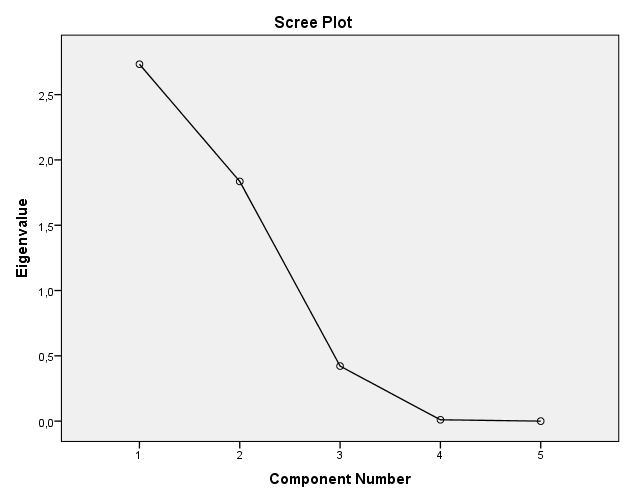


**Figure E:** eigenvalue plots (scree plots) for the climate PCA

|  | **Dimension** | | |
| --- | --- | --- | --- |
|  | **1** | **2** | **3** |
| National Unemployment rate_ma3 | .955 |  |  |
| National Growth rate_ma3 |  |  | .982 |
| GDP per capita_ma3 | -.779 |  |  |
| Inflation_ma3 |  | .982 |  |

**Table C:** PCA (2^nd^ layer) solution for the economy variables. A 3-dimension solution explains 93% of variance.

|  | **Dimension** | |
| --- | --- | --- |
|  | **1** | **2** |
| Rainfall_ma3 |  | -.851 |
| Temperature_ma3 | .991 |  |
| Monthly Max. Temperature_ma3 | .789 |  |
| Monthly Min. Temperature_ma3 | .917 |  |
| Annual Temperature Range_ma3 |  | .823 |

**Table D:** PCA (2^nd^ layer) solution for the climate variables. A 2-dimension solution explains 91.3% of variance

Thus, we have concluded with 3 orthogonal dimensions for the economy category (named below as **Economy Dimension 1, 2, 3**), and 2 dimensions for the climate category (named below as **Climate Dimension 1, 2**), see Table E. These dimensions were next subjected to CATREG as IV for the regression of male and female suicidal rates as DV.

| **Dimension** | **Corresponding variables** |
| --- | --- |
| Economy Dimension 1 - (E1) | National Unemployment rate_ma3, GDP per capita_ma3 (with opposing signs) |
| Economy Dimension 2 - (E2) | Inflation_ma3 (same sign with National Unemployment rate_ma3) |
| Economy Dimension 3 - (E3) | National Growth rate_ma3 (same sign with National Unemployment rate_ma3) |
| Climate Dimension 1 - (C1) | Temperature_ma3, Monthly Min. Temperature_ma3, Monthly Max. Temperature_ma3 (with same signs) |
| Climate Dimension 2 - (C2) | Rainfall_ma3, Annual Temperature Range_ma3 (with opposing signs; Annual Temperature Range_ma3 with same sign as dimension 1 variables) |

**Table E:** The formed economy and climate dimensions (after PCA) with their constituent quantities (see Table B).

*5c CATREG and Linear Multiple Regression*

As mentioned above, the previously obtained orthogonal dimensions were used as the IV for the regression analysis of suicidal rates.

Prior to the regression analysis, an inspection of fundamental statistical properties, histograms and scatterplots of the DV and IV revealed that all variables deviated significantly from normality showing higher absolute skewness values. This fact predisposes for the possibility of nonlinear relations between the DV and the IV, influential cases or outliers, thus hampering a simple linear model’s validity. Indeed, further examination of scatterplots between the DV and each IV revealed the possible existence of such phenomena. For this reason, at first hand, one could consider the possibility of making some typical numerical transformations (e.g. log-transform), especially for the DV (and Male Suicides in particular) as these take only positive values. This could also act as a remedy for the possible nonlinearities that were suspected. Another alternative, which is relieved from the need to explicitly specify each variable’s transformation function, would be the use of optimal transformation (scaling) within the framework of regression by employing the readily available CATREG algorithm which is implemented in the SPSS statistical processing software. CATREG uses optimal scaling of variables and alternating least squares together to provide an optimum regression solution and variable transformation with several variable quantification options (such as simple numerical quantification, ordinal, spline, etc.). A strong advantage of the procedure is its ability to optimally transform all selected variables at the same time. The obtained transformations need not have a closed-form expression although they might be approached as such in order to facilitate further interpretability and use of the regression model for purposes of prediction. The method has proved useful in raising deficiencies from nonlinearities and increasing the validity of solutions [3][6].

Following this, we decided to examine two different models of multiple regressions separately for each of the DV namely the Male Suicides and Female Suicides. For the male suicidality, the first model (M1m) is a log-linear multiple regression model in which the IV are all entered untransformed and the DV (the Male Suicides) is entered after a logarithmic transformation due to the original variable’s severe negative skewness, and the second model (M2m) is a CATREG model in which all IV variables are allowed to be optimally transformed after a mild spline ordinal quantification (2^nd^ order spline with 3 internal knots) to obtain both adequate transforming power and avoid possible overfitting, by maintaining a ratio ~10.5 of data points (~255) to degrees-of-freedom (5 [number of predictors] x (2 [order of spline] +3 [internal knots])) [7]. Overfitting in a model reduces the prediction error but with a hard penalty, namely the increased complexity in terms of the number of parameters [8][6][9][10]. Actually, it risks describing the inherent noise and uncertainty in data, than the underlying general behavior of the associated quantities. As it is always possible to identify a higher-order polynomial function to fit our data, in general we need to compromise a higher prediction error in favor of a simpler function. Indeed, in our study, it can be shown that with either increasing the order of the spline and/or the number of knots, it will be the case that the prediction error will be further reduced, and the *R^2^* and the *adjusted R^2^* will be reduced accordingly. However, we want to avoid such a situation, in order to highlight the inherent and more generalizable properties of relationships between the investigated variables. The DV was kept untransformed in order to keep predicted values in direct equivalence to raw data. For the female suicidality, model M1f is a linear multiple regression model in which both the DV and the IV are all entered untransformed (the Female Suicides pdf is actually close to normal), and model M2f is again a CATREG model with the same specifications as in M2m. The examination of these models will allow a comparison of their validity and predictive capacity. Finally, and in order to show the value of the combined use of both of these two categories as predictors (omnibus approach), we also conducted CATREG analysis for Male Suicides and Female Suicides similarly to models M2m and M2f, but including only the economy or climate dimensions as IV (segregated approach). Thus the respective models were coded as Mme (Male Suicides vs economy factors), Mmc (Male Suicides vs climate factors), Mfe (Female Suicides vs economy factors), Mfc (Female Suicides vs climate factors).

***5c-1. Males***

*5c-1a Model M1m*

The results returned an R=0.669 and R-square=0.447 (adjusted R^2^=0.436) with a standard error of estimate equal to 0.166. The details of the results are shown in table F below.

|  | **Unstandardized Coefficients** | | **Standardized Coefficients** | **t** | ***p*** | **Collinearity Statistics** | |
| --- | --- | --- | --- | --- | --- | --- | --- |
|  | **B** | **Std. Error** | **Beta** |  |  | **Tolerance** | **VIF** |
| (Constant) | 1.258 | .010 |  | 119.818 | .000 |  |  |
| **Economy Dimension 1** | .113 | .014 | .489 | 8.025 | .000 | .608 | 1.645 |
| **Economy Dimension 2** | .054 | .012 | .234 | 4.510 | .000 | .836 | 1.196 |
| **Economy Dimension 3** | .036 | .011 | .162 | 3.275 | .001 | .918 | 1.089 |
| **Climate Dimension 1** | -.131 | .011 | -.589 | -11.724 | .000 | .894 | 1.118 |
| **Climate Dimension 2** | -.024 | .014 | -.110 | -1.746 | .082 | .566 | 1.767 |

**Table F:** Model’s M1m regression coefficients and variance statistics

These values were obtained after removing 1 outlier case (Greece for the year 2002) which was detected by inspecting the standardized predicted values vs. standardized residuals.

All coefficients are statistically significant except for the coefficient of the 2^nd^ Climate Dimension (Rainfall and Annual Temperature Range). VIF values are kept low enough (<2.5) showing no mandatory collinearity issues. From the standardized beta coefficients, the major and almost equally contributing factors are the 1^st^ Economy Dimension (National Unemployment rate_ma3 and GDP per capita_ma3) and the 1^st^ Climate Dimension (Temperature_ma3) while the rest of the economy dimensions (Inflation_ma3 and National Growth rate_ma3) show a statistically significant but rather negligible impact compared to the two major contributors. In more detail, the obtained regression model is described as (the IVs are as in Table B, bold typeface indicates statistical significance at the 0.05 level):

MSu = **1.258 + 0.113*E1 + 0.054*E2 + 0.036*E3 - 0.131*C1** - 0.024*C2 (eq. 1)

The obtained scatterplot of standardized predicted values vs. standardized residuals (Figure F) shows random distribution of residuals without clear signs of nonlinearity or strong heteroscedasticity although some cases seem more isolated from the main swarm of points. The residuals’ Q-Q plot (Figure G) still remains close to normality. The model’s predictive capacity is assessed in terms of both the *adjusted R^2^* value and the *Stein’s adjusted R^2^* value which is 0.421.


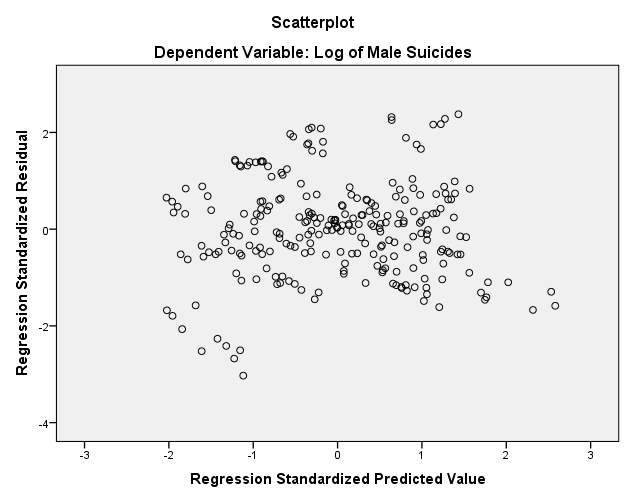


**Figure F:** Scaterplot of Predicted values vs. Residuals


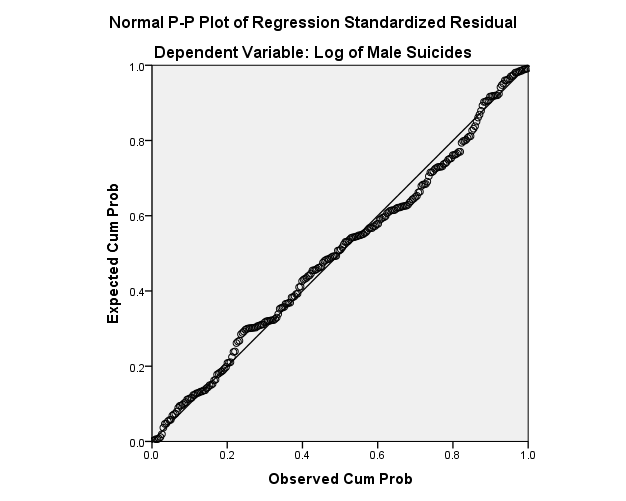


**Figure G:**  *Residuals’ normal P-P plot*

*5c-1b Model M2m*

The results returned an R=0.790 and R-square=0.624 (adjusted R^2^=0.602) with a standard error of estimate equal to 0.376. The details of the results are shown in table G below.

|  | Standardized Coefficients | | df | F | *p* |
| --- | --- | --- | --- | --- | --- |
|  | Beta | Std. Error |  |  |  |
| **Economy Dimension 1** | .685 | .110 | 3 | 38.441 | .000 |
| **Economy Dimension 2** | .261 | .054 | 3 | 23.219 | .000 |
| **Economy Dimension 3** | .147 | .057 | 4 | 6.717 | .000 |
| **Climate Dimension 1** | -.681 | .052 | 2 | 171.385 | .000 |
| **Climate Dimension 2** | -.249 | .183 | 2 | 1.856 | .159 |

**Table G:** Results of model M2m (all IV variables are allowed to be optimally transformed after a mild spline ordinal quantification that is 2^nd^ order spline with 4 internal knots, to obtain both appropriate transforming and avoid possible overfitting while the DV was kept untransformed in order to keep predicted values in directly equivalence to raw data)

| 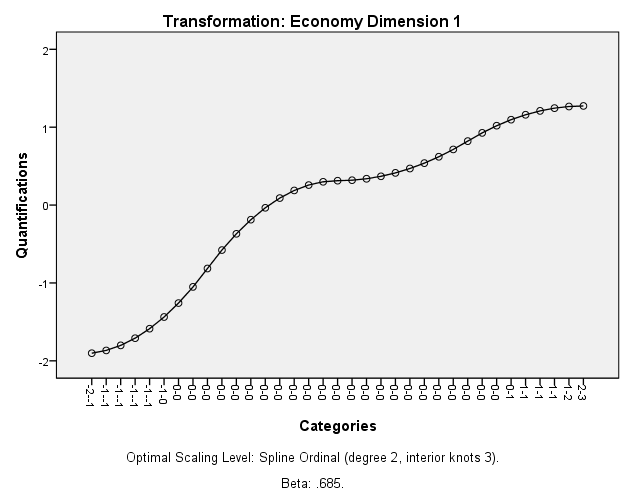 | 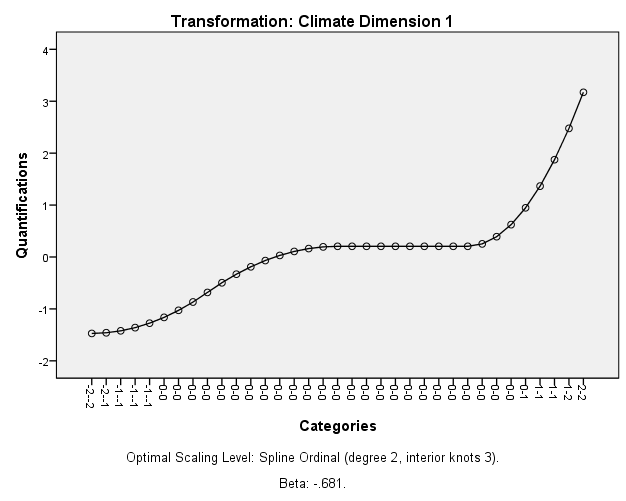 |
| --- | --- |
| 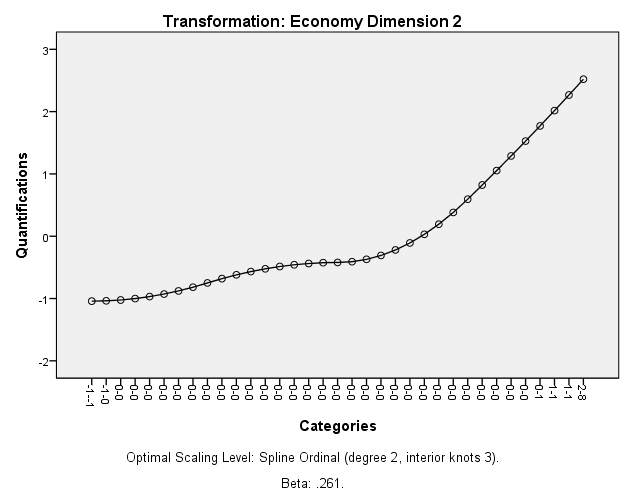 | 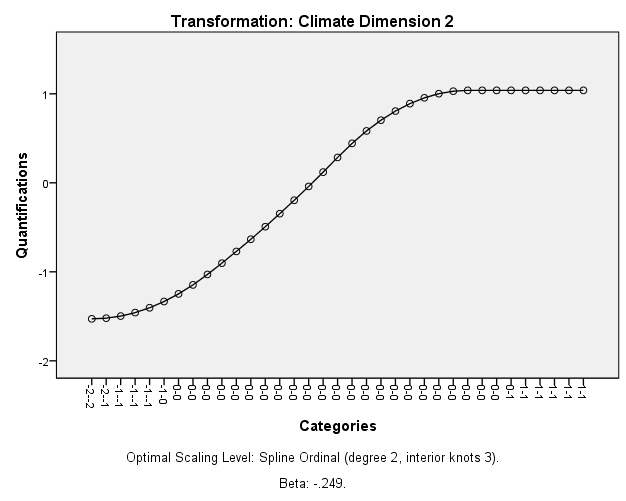 |
| 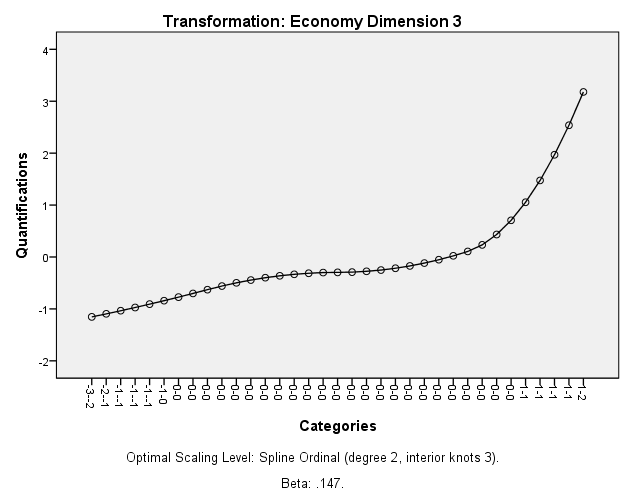 |  |

**Figure H:** Transformation plots of Economy and Climate Dimensions after optimal scaling with CATREG for model M2m

These results have been obtained after removing 3 outlier cases (Slovakia for the years 2006-2007 and Montenegro for the year 2006) which were detected by inspecting the standardized predicted values vs. standardized residuals.

The CATREG procedure reveals strong nonlinearities that are addressed by appropriate transformation of the IV variables (Figure H). As for model M1m, all coefficients are statistically significant except for the coefficient of the 2^nd^ Climate Dimension (Rainfall_ma3 and Annual Temperature Range_ma3). VIF values (after performing a linear regression with the optimally transformed variables) are kept low enough (<2.5) showing no mandatory collinearity issues. From the standardized beta coefficients, the major and equally contributing factors are the 1^st^ Economy Dimension (National Unemployment rate_ma3 and GDP per capita_ma3) and the 1^st^ Climate Dimension (Temperature_ma3). However, in contrast to model M1m, the contribution of Inflation and National Growth rate_ma3 are not negligible. In more detail, the obtained regression model with optimally quantified (standardized and transformed) variables is described as:

MSu = **0.685*E1 + 0.261*E2 + 0.147*E3 - 0.681*C1** - 0.249*C2 (eq. 2)

The obtained scatterplot of standardized predicted values vs. standardized residuals (Figure I) shows a more random distribution of residuals compared to model M1m, again without clear signs of nonlinearity or heteroscedasticity, but with a more uniform swarm of points. Despite a slight deviation from pure normality, which shall rather be attributed to the fact that the transformation of the IVs only could not fully compensate for not transforming the DV also, the residuals’ Q-Q plot (Figure J) remains close to normality. The model’s predictive capacity is assessed in terms of both the *adjusted R^2^* value and the *Stein’s adjusted R^2^* value which is 0.603, which are clearly superior to the respective M1m model’s figures.


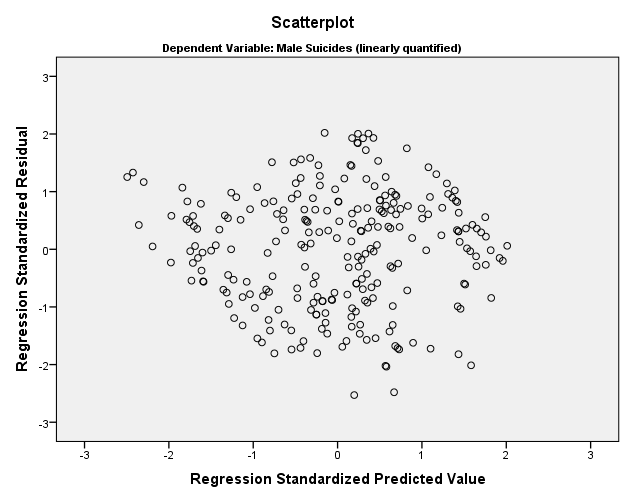


**Figure I:** Scaterplot of Predicted values vs. Residuals


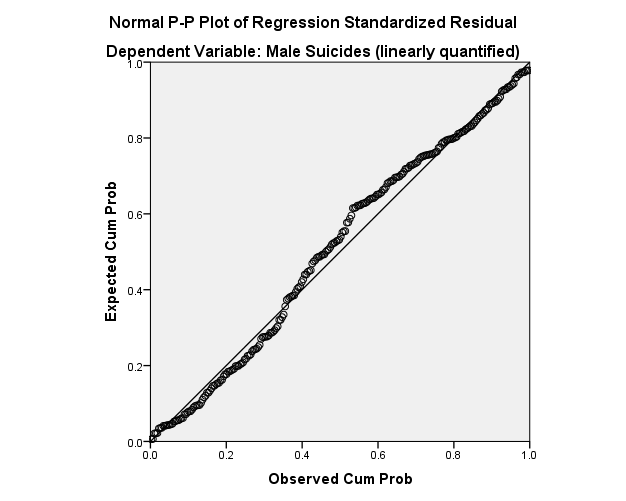


**Figure J:** Residuals’ normal P-P plot

In conclusion, the model M2m with optimally transformed IVs shows superior fitting and predictive power compared to model M1m and, thus, should be chosen as more appropriate for the description of the relation between male suicidal rates and economy/climate dimensions. (Equation 2) can be reformulated in terms of emerging economy and climate factors as:

**Male suicidality = (economy) + (climate)** (eq. 3)

with:

**(economy) = 0.685*(NU_ma3 & GDP_ma3) - 0.261*(Infl_ma3) + 0.147*(NG_ma3)**

**(climate) = - 0.681*(Temp_ma3, MmT_ma3, MMT_ma3)** - 0.249*(Rfl_ma3, ATR_ma3)

The respective standardized beta coefficients are .804 for the **(economy)** emerging factor and .720 for the **(climate)** factor, which shows that the two factors may be considered of equivalent importance.

*5c-1c Model Mme*

The results returned an R=0.501 and R-square=0.251 (adjusted R^2^=0.236) with a standard error of estimate equal to0.749. The details of the results are shown in table H below.

|  | Standardized Coefficients | | df | F | *p* |
| --- | --- | --- | --- | --- | --- |
|  | Beta | Bootstrap (1000) Estimate of Std. Error |  |  |  |
| **Economy Dimension 1** | .292 | .051 | 2 | 32.942 | .000 |
| **Economy Dimension 2** | .222 | .070 | 2 | 10.122 | .000 |
| **Economy Dimension 3** | .263 | .078 | 1 | 11.430 | .001 |

**Table H:** Results of model Mme (use of economic variables only as IV)

| 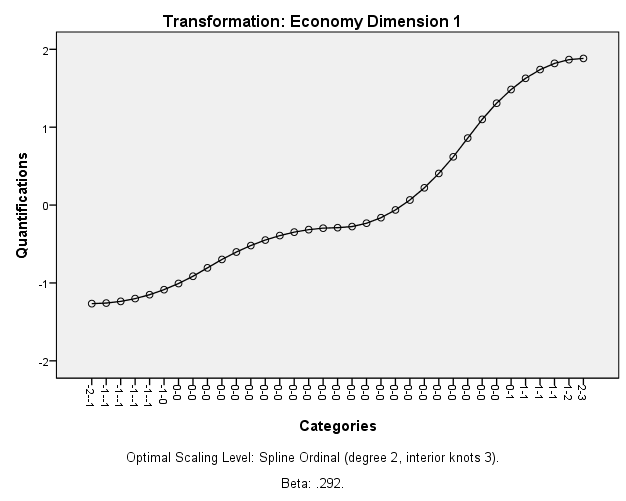 |
| --- |
| 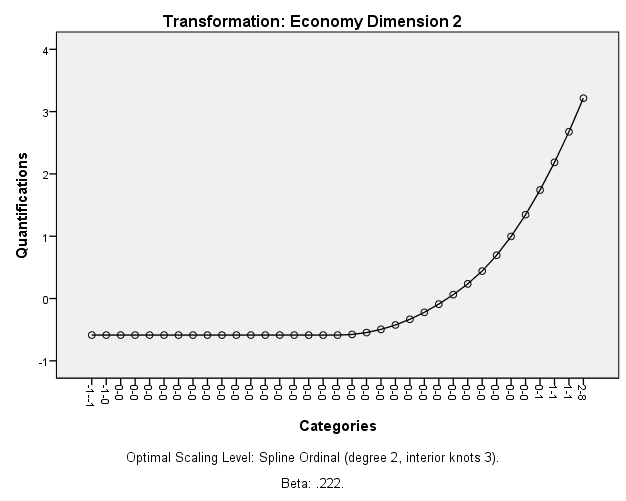 |
| 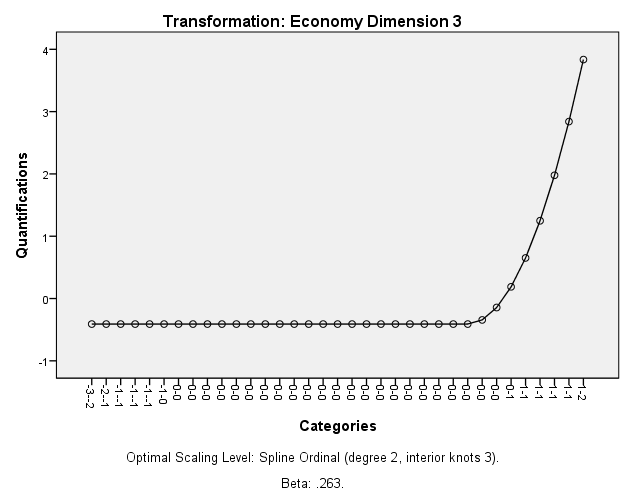 |

**Figure K:** Transformation plots of Economy Dimensions after optimal scaling with CATREG for model Mme

In this model, the CATREG procedure also reveals strong nonlinearities that are addressed by appropriate transformation of the IV variables (Figure K). However the model’s performance is marginal. One case was close to be labeled as outlier (Slovakia for the year 2007) by inspecting the standardized predicted values vs. standardized residuals. All coefficients are statistically significant and of equivalent contribution. In more detail, the obtained regression model with optimally quantified (standardized and transformed) variables is described as:

MSu = **0.292*E1 + 0.222*E2 + 0.263*E3** (eq. 4)

The obtained scatterplot of standardized predicted values vs. standardized residuals (Figure L) still shows a random distribution of residuals but with less uniformity. The residuals’ Q-Q plot (Figure M) shows a slight deviation from pure normality, which again could be due to incomplete compensation by the transformation of IV. The model’s predictive capacity is assessed in terms of both the *adjusted R^2^* value and the *Stein’s adjusted R^2^* value which is0.229, which are clearly inferior to the respective M1m and M2m models’ figures.


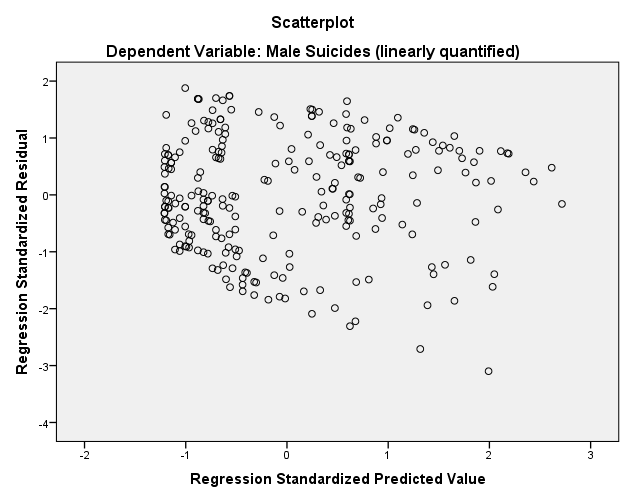


**Figure L:** Scaterplot of Predicted values vs. Residuals


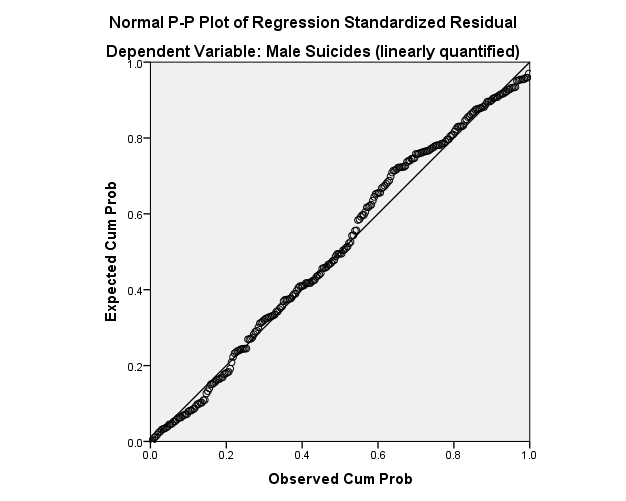


**Figure M:** Residuals’ normal P-P plot

*5c-1d Model Mmc*

The results returned an R=0.614 and R-square=0.376 (adjusted R^2^=0.363) with a standard error of estimate equal to 0.624. The details of the results are shown in table I below.

|  | Standardized Coefficients | | df | F | *p* |
| --- | --- | --- | --- | --- | --- |
|  | Beta | Bootstrap (1000) Estimate of Std. Error |  |  |  |
| **Climate Dimension 1** | -.520 | .044 | 2 | 142.486 | .000 |
| **Climate Dimension 2** | .328 | .042 | 4 | 61.493 | .000 |

**Table I:** Results of model Mmc (use of climate variables only as IV)

| 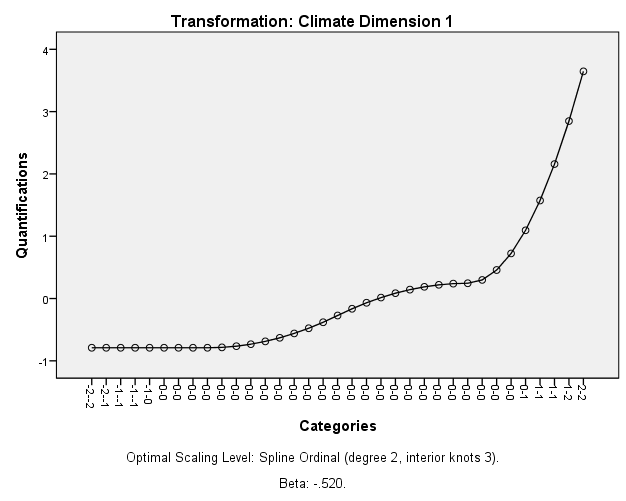 |
| --- |
| 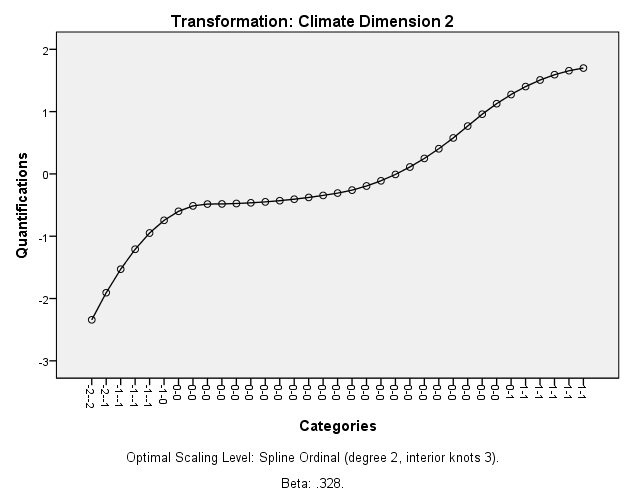 |

**Figure N:** Transformation plots of Climate Dimensions after optimal scaling with CATREG for model Mmc

In this model, the CATREG procedure also reveals strong nonlinearities that are addressed by appropriate transformation of the IV variables (Figure N). The model’s *R^2^* is just 0.376 and the *adjusted R^2^* is 0.363 which are clearly better than model’s Mme, showing better fitting capacity of the climate dimensions compared to economy, but the performance still remains rather low. Both coefficients are statistically significant but the 1^st^ Climate Dimension (Temperature_ma3) is of larger contribution. The obtained regression model with optimally quantified (standardized and transformed) variables is described as:

MSu = **- 0.520*C1 + 0.328*C2** (eq. 5)

The obtained scatterplot of standardized predicted values vs. standardized residuals (Figure O) still shows a random distribution of residuals but with some discrete islands of points. The residuals’ Q-Q plot (Figure P) however shows strong deviations from normality, as the transformations of IVs prove inadequate to fully recover linearity and asymmetry. The model’s predictive capacity is assessed in terms of both the *adjusted R^2^* value and the *Stein’s adjusted R^2^* value which is 0.364, clearly inferior to the respective M1m and M2m models’ figures.


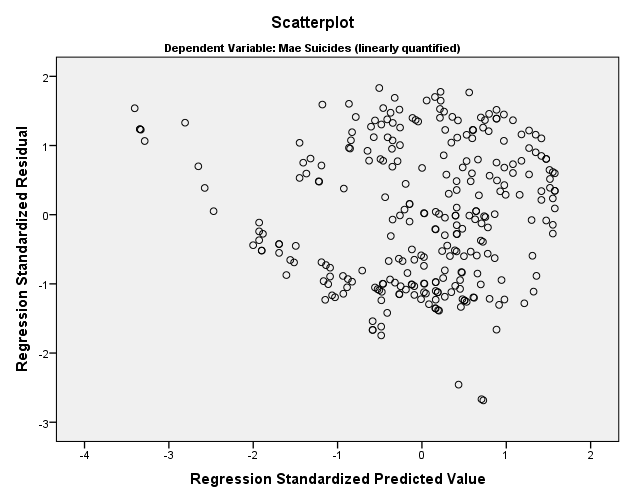


**Figure O:** Scaterplot of Predicted values vs. Residuals


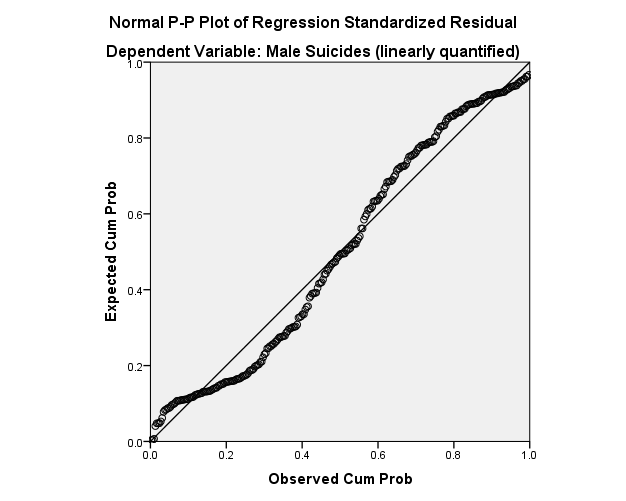


**Figure P:**  *Residuals’ normal P-P plot*

All in all, the singular models Mme and Mmc are of apparently lower fitting and prediction capabilities compared to the omnibus M1m and M2m models, highlighting the value of the combined set of predictors (economy plus climate) for the description of male suicidal rates.

***5c-2 Females***

*5c-2a Model M1f*

The results returned an R=0.531 and R-square=0.282 (adjusted R^2^=0.268) with a standard error of estimate equal to 1.879. The details of the results are shown in table J below.

|  | Unstandardized Coefficients | | Standardized Coefficients | t | *p* | Collinearity Statistics | |
| --- | --- | --- | --- | --- | --- | --- | --- |
|  | B | Std. Error | Beta |  |  | Tolerance | VIF |
| (Constant) | 5.218 | .119 |  | 43.978 | .000 |  |  |
| **Economy Dimension 1** | .491 | .159 | .213 | 3.078 | .002 | .608 | 1.644 |
| **Economy Dimension 2** | -.121 | .135 | -.053 | -.897 | .370 | .838 | 1.194 |
| **Economy Dimension 3** | .179 | .124 | .081 | 1.444 | .150 | .918 | 1.090 |
| **Climate Dimension 1** | -1.170 | .126 | -.529 | -9.267 | .000 | .896 | 1.116 |
| **Climate Dimension 2** | -.192 | .155 | -.089 | -1.242 | .215 | .567 | 1.764 |

**Table J:** Results of model M1f (female suicidality as DV and the IV are all entered untransformed)

These results were obtained after removing 3 outlier cases (Slovakia for the years 2006-2007 and Montenegro 2006) which were detected by inspecting the standardized predicted values vs. standardized residuals. Statistical significance was obtained for the constant term, the first two Economy Dimensions and the first Climate Dimension. VIF values are again kept low (<2.5). The standardized beta coefficients show that the 1^st^ Climate Dimension (Temperature_ma3) contributes mostly to the regression, followed by the 1^st^ Economy Dimension (National Unemployment rate_ma3 and GDP per capita_ma3) which however shows a far small impact. Finally, the obtained regression model is described as:

FSu = **5.218 + 0.491*E1** - 0.121*E2 + 0.179*E3 **- 1.170*C1 -** 0.192*C2 (eq. 6)

Although the obtained scatterplot of standardized predicted values vs. standardized residuals (Figure Q) shows random distribution of residuals, the residuals’ Q-Q plot (Figure R) shows a significant deviation from normality *(Kolmogorov-Smirnoff statistic=0.067, p<0.01, Shapiro-Wilk statistic=0.973, p<0.01)*. The model’s predictive capacity is assessed in terms of both the *adjusted R^2^* value and the *Stein’s adjusted R^2^* value which is 0.249.


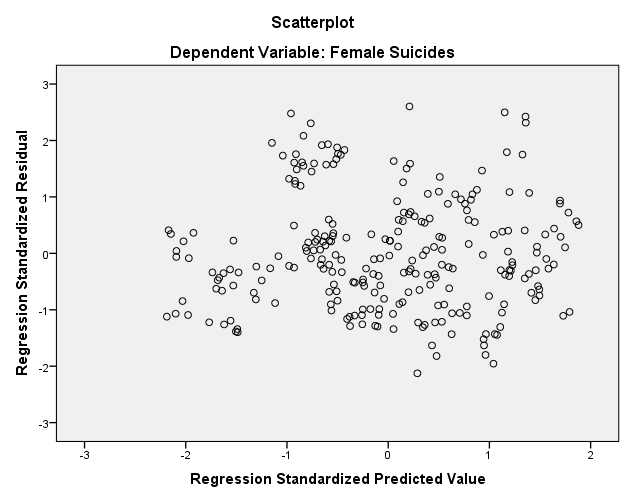


**Figure Q:** Scaterplot of Predicted values vs. Residuals


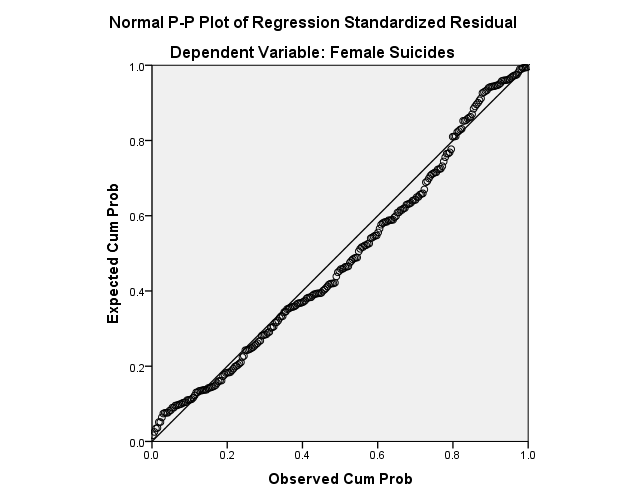


**Figure R:**  *Residuals’ normal P-P plot*

*5c-2b Model M2f*

The results returned an R=0.642 and R-square=0.412 (adjusted R^2^=0.390) with a standard error of estimate equal to0.588. The details of the results are shown in table K below.

|  | **Standardized Coefficients** | | **df** | **F** | *p* |
| --- | --- | --- | --- | --- | --- |
|  | **Beta** | **Std. Error** |  |  |  |
| **Economy Dimension 1** | .359 | .217 | 1 | 2.752 | .098 |
| **Economy Dimension 2** | -.230 | .067 | 2 | 11.766 | .000 |
| **Economy Dimension 3** | .040 | .109 | 2 | .133 | .876 |
| **Climate Dimension 1** | -.642 | .078 | 3 | 67.924 | .000 |
| **Climate Dimension 2** | -.356 | .276 | 1 | 1.661 | .199 |

**Table K:** Results of model M2f (all IV variables are allowed to be optimally transformed after a mild spline ordinal quantification that is 2^nd^ order spline with 3 internal knots, to obtain both appropriate transforming and avoid possible overfitting while the DV was kept untransformed in order to keep predicted values in directly equivalence to raw data)

| 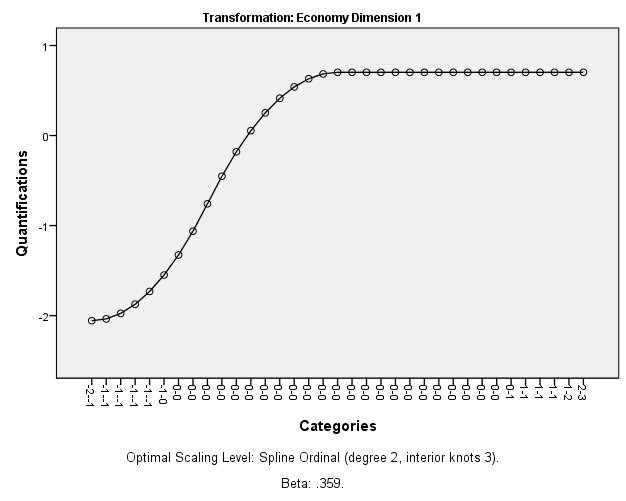 | 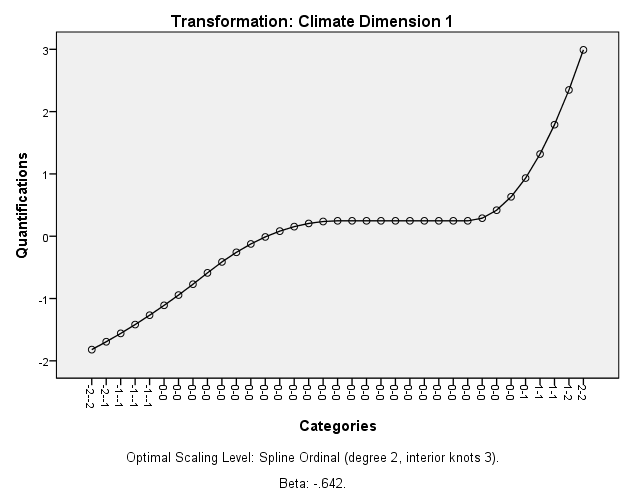 |
| --- | --- |
| 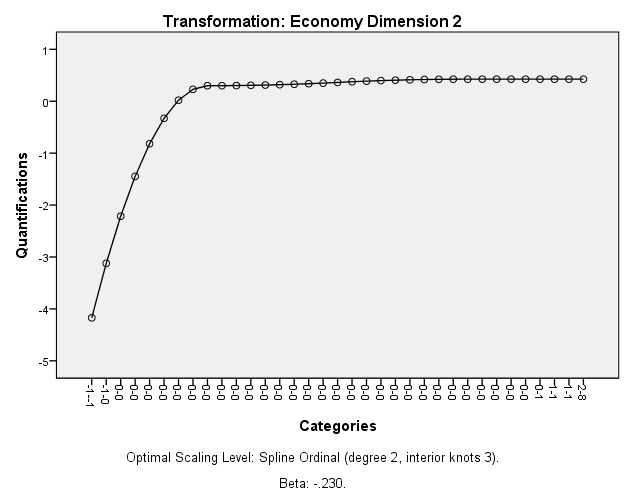 | 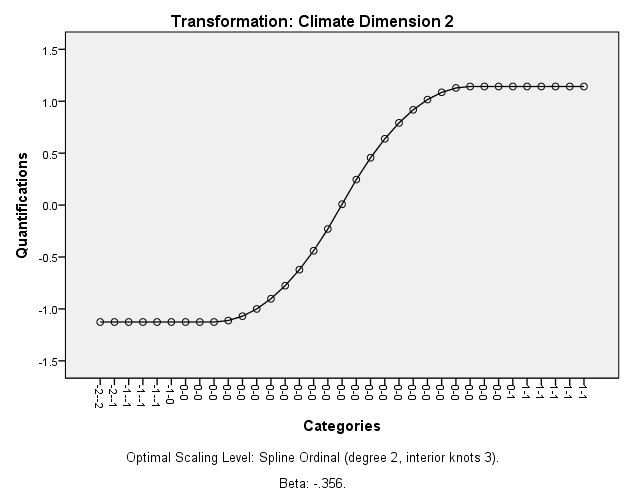 |
| 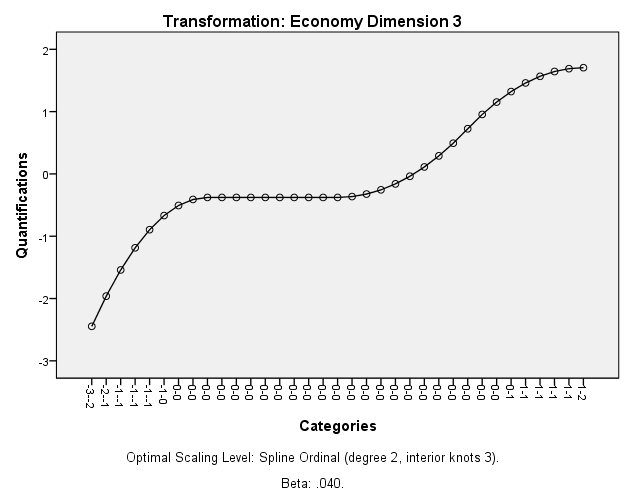 |  |

**Figure S:** Transformation plots of Economy and Climate Dimensions after optimal scaling with CATREG for model M2f

The CATREG procedure again reveals appropriate transformations of the IV variables (Figure S) to cope with nonlinearities, asymmetries, etc. The model’s *R^2^* is now raised to 0.412 and the *adjusted R^2^* is 0.390 which are considered sufficient (Cohen [11] suggests values higher than .25 to be considered “strong”) and are improved when compared to model M1f. One outlier case was removed (Montenegro 2006). The 2^nd^ Economy (Inflation_ma3) Dimension and the 1^st^ Climate Dimension (Temperature_ma3) are statistically significant, with the strongest contribution by the 1^st^ Climate Dimension. VIF values (after performing a linear regression with the optimally transformed variables) are kept low enough (<2.5) showing no mandatory collinearity issues. The obtained regression model with optimally quantified (standardized and transformed) variables is described as:

FSu = 0.359*E1 **- 0.230*E2** + 0.040*E3 **- 0.642*C1** - 0.356*C2 (eq. 7)

The obtained scatterplot of standardized predicted values vs. standardized residuals (Figure T) also shows a random distribution of residuals. However, in comparison to model M1f, the residuals are close to pure normality as shown by the Q-Q plot (Figure U) and by the respective statistical tests *(Kolmogorov-Smirnoff statistic=0.040, p=0.200, Shapiro-Wilk statistic=0.992, p=.212).* Finally, the model’s predictive capacity is again assessed in terms of both the *adjusted R^2^* value and the *Stein’s adjusted R^2^* value which is 0.390, which are improved over the figures of model M1f.


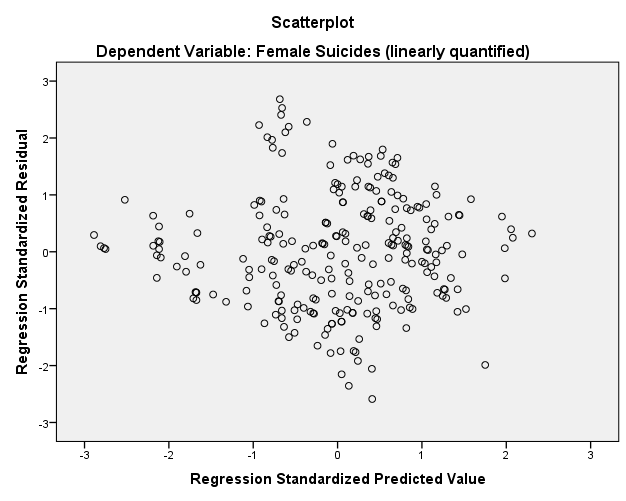


**Figure T:** Scaterplot of Predicted values vs. Residuals


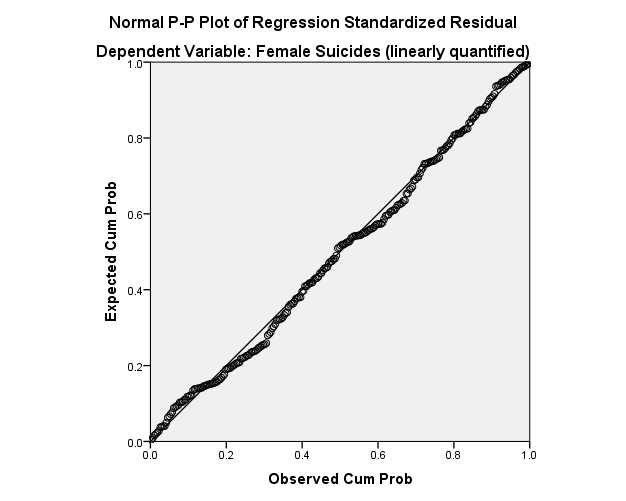


**Figure U:** Residual’s normal P-P plot

Thus, the model M2f with optimally transformed IVs shall be chosen over model M1f. (Equation 7) can be reformulated in terms of emerging economy and climate factors as:

**Female suicidality = (economy) + (climate)** (eq. 8)

with:

**(economy) =** 0.359*(NU_ma3 & GDP_ma3) **- 0.230*(Infl_ma3)** + 0.040*(NG_ma3)

**(climate) = - 0.642*(Temp_ma3, MmT_ma3, MMT_ma3)** - 0.356*(Rfl_ma3, ATR_ma3)

The respective standardized beta coefficients are .446 for the **(economy)** emerging factor and .723 for the **(climate)** factor, which shows a clearly bigger impact of the **(climate)** factor.

*5c-2c Model Mfe*

The results returned an R=0.343 and R-square=0.118 (adjusted R^2^=0.096) with a standard error of estimate equal to0.882. The details of the results are shown in table L below.

|  | **Standardized Coefficients** | | **df** | **F** | ***p*** |
| --- | --- | --- | --- | --- | --- |
|  | **Beta** | **Bootstrap (1000) Estimate of Std. Error** |  |  |  |
| **Economy Dimension 1** | -.120 | .097 | 3 | 1.525 | .209 |
| **Economy Dimension 2** | -.316 | .064 | 2 | 24.154 | .000 |
| **Economy Dimension 3** | .135 | .141 | 1 | .917 | .339 |

**Table L:** Results of model Mfe (use of economic variables only as IV)

| 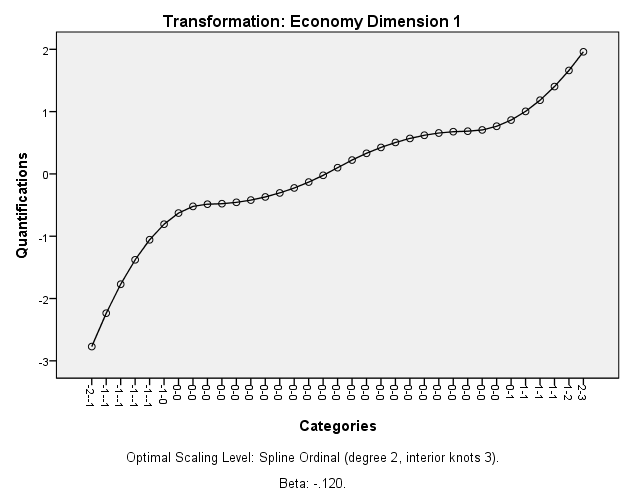 |
| --- |
| 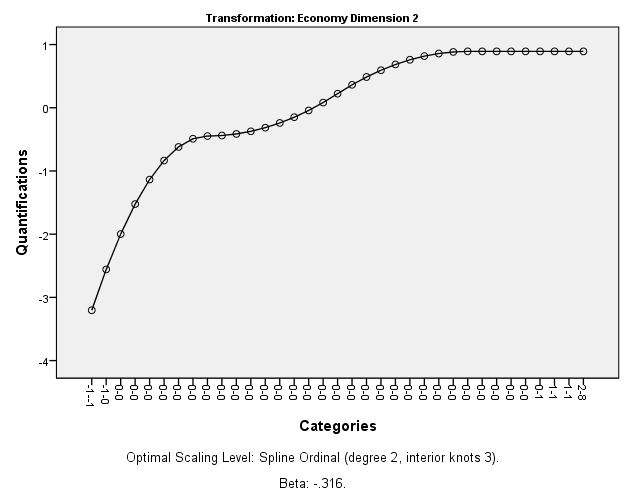 |
| 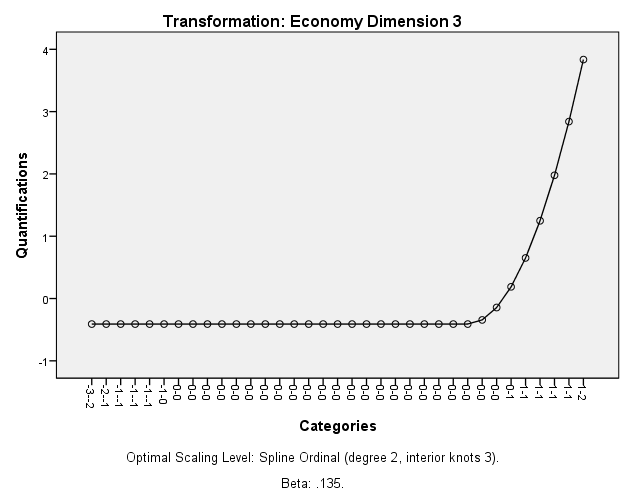 |

**Figure V:** Transformation plots of Economy Dimensions after optimal scaling with CATREG for model Mfe

In this model, the CATREG procedure also reveals strong nonlinearities (Figure V). However, the model’s *R^2^* is just 0.118 and the *adjusted R^2^* is 0.096 which are considered very low. Only the 2^nd^ Economy Dimension (Inflation_ma3) coefficient met statistical significance. The obtained regression model with optimally quantified (standardized and transformed) variables is described as:

FSu = - 0.120*E1 **- 0.316*E2** + 0.135*E3 (eq. 9)

The obtained scatterplot of standardized predicted values vs. standardized residuals (Figure W) shows a random distribution of residuals but the residuals’ Q-Q plot (Figure X) shows a rather strong deviation from normality, which goes with the low fitting performance. The model’s predictive capacity in terms of both the *adjusted R^2^* value and the *Stein’s adjusted R^2^* value is very low also (0.096 and 0.092 respectively). Such values are clearly inferior to the respective M1f and M2f models’ figures.


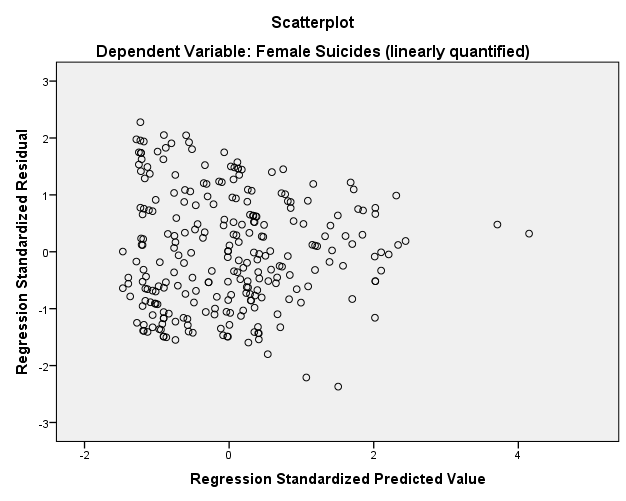


**Figure W:** Scaterplot of Predicted values vs. Residuals


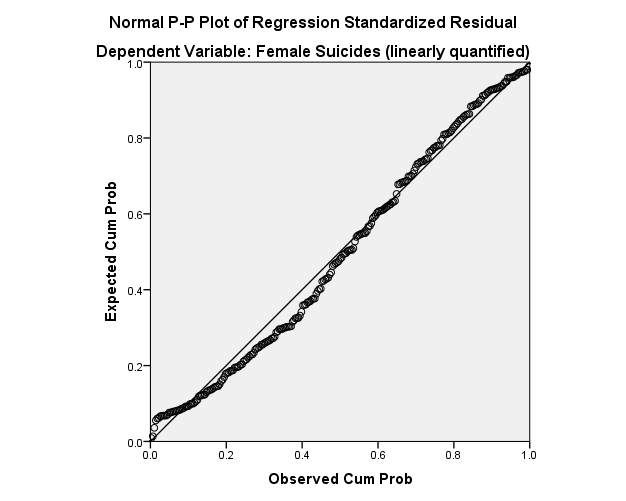


**Figure X:**  *Residual’s normal P-P plot*

*5c-2d Model Mfc*

The results returned an R=0.535 and R-square=0.286 (adjusted R^2^=0.276) with a standard error of estimate equal to0.714. The details of the results are shown in table M below.

|  | **Standardized Coefficients** | | **df** | **F** | ***p*** |
| --- | --- | --- | --- | --- | --- |
|  | **Beta** | **Bootstrap (1000) Estimate of Std. Error** |  |  |  |
| **Climate Dimension 1** | -.527 | .035 | 3 | 227.464 | .000 |
| **Climate Dimension 2** | -.102 | .108 | 1 | .900 | .344 |

**Table M:** Results of model Mfc (use of climate variables only as IV)

| 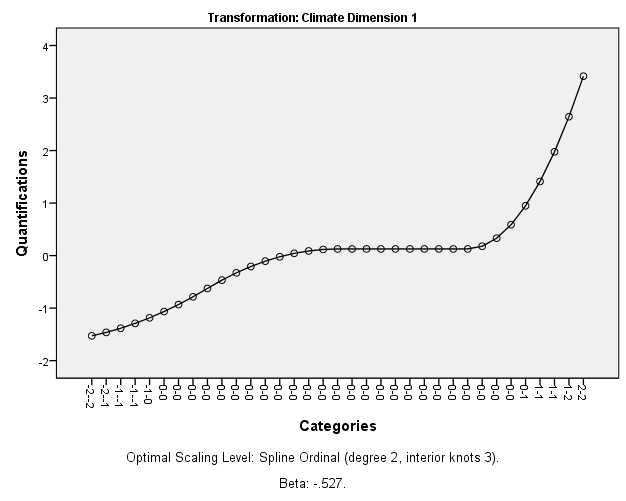 |
| --- |
| 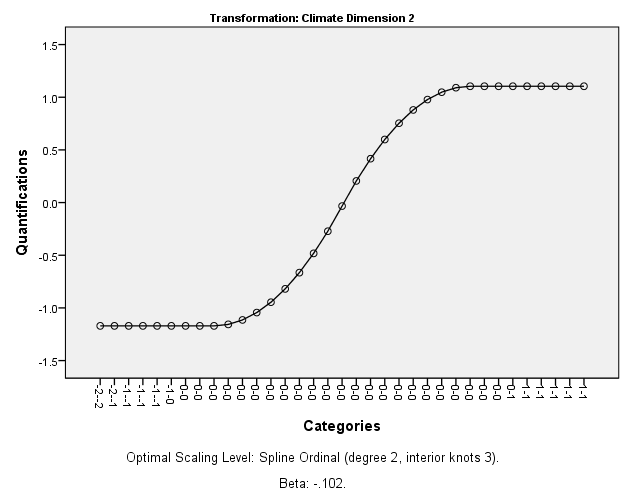 |

**Figure Y:** Transformation plots of Climate Dimensions after optimal scaling with CATREG for model Mfc

Again, the CATREG procedure reveals strong nonlinearities (Figure Y) that are addressed by appropriate transformation of the IV variables. The model’s *R^2^* is just 0.286 (marginal). Only the 1^st^ Climate Dimension (Temperature_ma3) coefficient is statistically significant. The obtained regression model with optimally quantified (standardized and transformed) variables is described as:

FSu = **- 0.527*C1** - 0.102*C2 (eq. 10)

The obtained scatterplot of standardized predicted values vs. standardized residuals (Figure Z) still shows a less random distribution of residuals and the residuals’ Q-Q plot (Figure AA) shows strong deviations from normality which also aligns with low fitting performance. The model’s predictive capacity in terms of both the *adjusted R^2^* value and the *Stein’s adjusted R^2^* value is also marginal (0.276 and 0.261 respectively) and remains inferior to the respective M1f and M2f models’ figures.


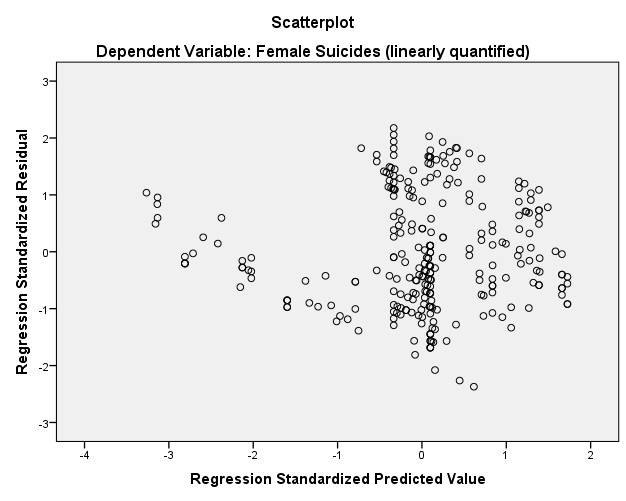


**Figure Z:** Scaterplot of Predicted values vs. Residuals


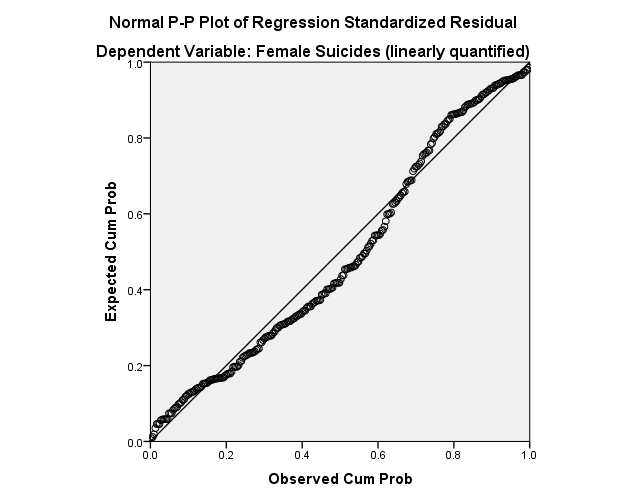


**Figure AA:** *Residuals’ normal P-P plot*

1. **Summary of results**

For males, the results of the regression analysis with all the independent variables optimally transformed after a mild spline ordinal quantification (that is 2^nd^ order spline with 3 internal knots, to obtain both appropriate transforming and avoid possible overfitting) while the dependent variables were kept untransformed (in order to keep predicted values in directly equivalence to raw data) returned an R=0.790 and R-square=0.624 (adjusted R^2^=0.602) with a standard error of estimate equal to 0.376. This model explained 62.4% of the variability of observed male suicidal rates with the combination of all available variables. The respective models with economic variables alone could explain up to 25.1% and climate variables alone up to 37.6%.

All economy variables (dimensions 1-3) but only one climate (dimension 1) contributed significanlty to this model. All economic variables had beta coefficients of the same (positive) sign suggesting that male suicides correlate with high unemployment rate in the frame of high growth rate and inflation and low GDP per capita. The negative sign of the beta coefficient concerning the climate variable suggests that male suicides correlate with low temperature both maximum and minimum (overall cold climate).

For females, the results of a similar analysis returned an R=0.642 and R-square=0.412 (adjusted R^2^=0.390) with a standard error of estimate equal to 0.583. This model explained 41.7% of the variability of observed female suicidal rates with the combination all available variables. Economic variables alone could explain up to 11.8% and climate variables alone up to 28.6%.

From economic variables only dimension 2 and from climate variables only dimension 1 contributed significantly to the model. The negative sign of the beta concerning the economic variable suggests a negative correlation between female suicidality and inflation while the negative sign of the beta coefficient concerning the climate variable suggests that male suicides correlate with low temperature both maximum and minimum (overall cold climate).

These models suggest that distinct economic but the same climate variables have an effect on male and female suicides. The selected models have good predictive capacity and fit the data well.

**References**

[1] B. S. Everitt, S. Landau, and M. Leese, *Cluster Analysis*, 4th ed. Wiley Publishing, 2009.

[2] H. Harman, *Modern Factor Analysis*, Third Edition, Revised edition. Chicago: University of Chicago Press, 1976.

[3] A. Zacharakis, K. Pastiadis, and J. D. Reiss, “An interlanguage study of musical timbre semantic dimensions and their acoustic correlates,” *Music Percept.*, vol. 31, no. 4, pp. 339–358, 2014.

[4] J. J. Meulman, “Optimal scaling methods for multivariate categorical data analysis,” SPSS, Chicago, White Paper, 2003.

[5] R. D. Ledesma and P. Valero-Mora, “Determining the number of factors to retain in EFA: An easy-to-use computer program for carrying out parallel analysis,” *Pract. Assess. Res. Eval.*, vol. 12, no. 2, pp. 1–11, 2007.

[6] J. J. Meulman, A. J. Van der Kooij, and W. J. Heiser, “Principal components analysis with nonlinear optimal scaling transformations for ordinal and nominal data,” in *The SAGE Handbook of Quantitative Methodology for the Social Sciences*, D. Kaplan, Ed. Thousand Oaks: Sage Publications, Inc., 2004, pp. 49–70.

[7] C. R. Wilson VanVoorhis and B. L. Morgan, “Understanding power and rules of thumb for determining sample sizes,” *Tutor. Quant. Methods Psychol.*, vol. 3, no. 2, pp. 43–50, 2007.

[8] A. J. van der Kooij, “Prediction accuracy and stability of regression with optimal scaling transformations,” Doctoral thesis, Leiden University, Faculteit der Sociale Wetenschappen, Leiden, 2007.

[9] S. Fortmann-Roe, “Accurately Measuring Model Prediction Error.” [Online]. Available: http://scott.fortmann-roe.com/docs/MeasuringError.html. [Accessed: 17-Nov-2015].

[10] F. Ye and Z. Zhao, “What You See May Not be What You Get – A Brief Introduction to Overfitting,” presented at the Cancer Biostatistics Workshop, Vanderbilt University, Nashville, TN, 2010.

[11] J. Cohen, “A power primer.,” *Psychol. Bull.*, vol. 112, no. 1, p. 155, 1992.
